# Supplementary material for: The Homogeneous Gas-Phase Formation Mechanism of PCNs from Cross-Condensation of Phenoxy Radical with 2-CPR and 3-CPR: A Theoretical Mechanistic and Kinetic Study
Source: Int J Mol Sci. 2022 May 24;23(11):5866. doi: 10.3390/ijms23115866 (PMC9180072; doi:10.3390/ijms23115866)
Supplement: Supplementary file 1 [file ijms-23-05866-s001.zip › ijms-1690319-supplementary.pdf]

Ninety-two pages

Contains computational details, one figure and five tables

Figure S1. The schematic energy profiles of the H abstraction from CPs by OH radical. ΔH is calculated at 0 K.

Table S1. The potential barriers ΔE (in kcal/mol) and the reaction heats ΔH (in kcal/mol) of elementary reactions involved in the formation of PhR, 2-CPR, and 3-CTPR from Phenol, 2-CP, and 3-CP, respectively. ΔH is calculated at 0 K.

Table S2. Imaginary frequencies (in cm^{-1}), zero-point energies (ZPE, in a.u.), and total energies (in a.u.) for the transition states involved in the formation of PCNs from cross–condensation of PhR with 2-CPR/3-CPR.

Table S3. CVT/SCT rate constants for the formation PCNs from cross–condensation of PhR with 2-CPR/3-CPR over the temperature range of 600–1200 K (units are s^{-1} and cm^3 $molecule^{-1}$ s^{-1} for unimolecular and bimolecular reactions, respectively).

Table S4. Cartesian coordinates for the transition states involved in PCN formation from cross-condensation of PhR with 2-CPR/3-CPR.

Table S5. Cartesian coordinates for the intermediates and products involved in PCN formation from cross–condensation of PhR with 2-CPR/3-CPR.

Computational details

Transition state: Transition state theory suggests that the conversion of reactants into products requires a high energy activation state, which is known as the transition state. All energies mentioned in this paper include zero-point energy (ZPE) correction.

Potential barriers (△*E*): The potential barrier is obtained by subtracting the energy of the reactants from the energy of the transition state.

Reaction heats (△*H*): The reaction heat is obtained by subtracting the energy of the products from the energy of the reactants.

The calculation steps:

We model all the required configurations in GaussView and then enter the appropriate computational commands to optimize them in Gaussian 09 program package [39] to the most stable state. We first selected the reactants and products for optimization, then designed the transition states and performed optimization calculations based on the structures of the optimized reactants and products.

Intrinsic reaction coordinate (IRC) calculations [40] were used to verify the reaction coordinates from the transition state towards the reactants and products respectively. Specifically, we selected 40 non-stationary points near the transition state of the minimum energy path for frequency calculations, including 20 points on the reactant side and 20 points on the product side. The reaction coordinate range under consideration –1.0~1.0 bohr. The SRANGE required to specify the reaction coordinate limits is selected from –1.5~1.5. The variable keyword in the Polyrate 9.7 program, SSTEP, which specifies the step size along the mass-scaled MEP and is identified as 0.05. Parameters such as the hessian matrixes, energy data and coordinates we need can be obtained from the Gaussian 09 output file and are input in the Polyrate 9.7 program [42] automatically by our self-compile program. The reaction rate constants in the temperature range 600–1200 K are available in the output file of Polyrate. Rate constants in this study were calculated using the canonical vibrational transition state theory (CVT) with small-curvature tunneling (SCT) correction. Through All the data calculated in the article are obtained from this series of calculations.

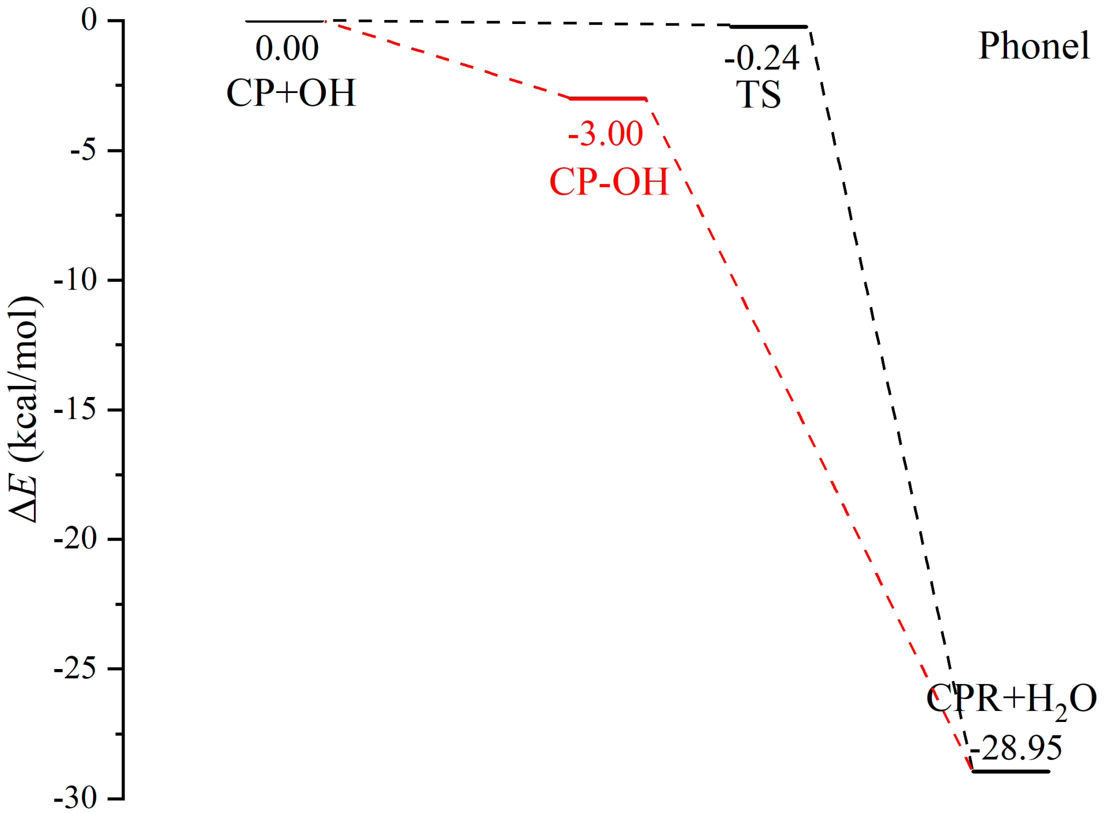

Figure S1. The schematic energy profiles of the H abstraction from CPs by OH radical. Δ*H* is calculated at 0 K. [42]

Table S1. The potential barriers ΔE (in kcal/mol) and the reaction heats ΔH (in kcal/mol) of elementary reactions involved in the formation of PhR, 2−CPR, and 3−CTPR from Phenol, 2−CP, and 3−CP, respectively. ΔH is calculated at 0 K.

Reaction	ΔE	ΔH	Reference
phenol → phenoxy + H	–	83.95	[42]
phenol + H → phenoxy + H_2	11.73	−3.98	[42]
phenol + OH → phenoxy + H_2O	−0.24	−28.95	[42]
phenol + O(^{3}P) → phenoxy + OH	5.45	−13.32	[42]
phenol + Cl → phenoxy + HCl	−8.28	−16.93	[42]
2-CP → 2-CPR + H	–	85.91	[31]
2-CP + H → 2-CPR + H_2	13.80	−12.01	[31]
2-CP + OH → 2-CPR + H_2O	3.20	−26.91	[31]
2-CP + O(^{3}P) → 2-CPR + OH	7.51	−11.35	[31]
2-CP + Cl → 2-CPR + HCl	−2.32	−14.96	[31]
3-CP → 3-CPR + H	–	84.99	[32]
3-CP + H → 3-CPR + H_2	12.51	−12.94	[32]
3-CP + OH → 3-CPR + H_2O	0.17	−27.81	[32]
3-CP + O(^{3}P) → 3-CPR + OH	8.20	−12.04	[32]
3-CP + Cl → 3-CPR + HCl	−5.87	−15.66	[32]

Table S2. Imaginary frequencies (in cm^{-1}), zero-point energies (ZPE, in a.u.), and total energies (in a.u.) for the transition states involved in the formation of PCNs from cross-condensation of PhR with 2−CPR/3−CPR.

Transition states	Imaginary frequencies	ZPE	Total energies
TS1	529i	0.18026	−1073.19492
TS2	734i	0.17964	−1073.12998
TS3	404i	0.16949	−959.85945
TS4	620i	0.16778	−959.79962
TS5	588i	0.18059	−1073.17114
TS6	727i	0.17848	−1073.12652
TS7	532i	0.16971	−959.86908
TS8	737i	0.16862	−959.80297
TS9	540i	0.18049	−1073.18320
TS10	718i	0.17927	−1073.12518
TS11	708i	0.16901	−959.85597
TS12	637i	0.16700	−959.79674
TS13	629i	0.18031	−1073.17467
TS14	674i	0.17831	−1073.11856
TS15	566i	0.16957	−959.85809
TS16	716i	0.16764	−959.79706
TS17	538i	0.18040	−1073.19800
TS18	702i	0.17949	−1073.13284
TS19	469i	0.16924	−959.86862
TS20	682i	0.16798	−959.79784
TS21	531i	0.18079	−1073.18483
TS22	688i	0.17925	−1073.12766
TS23	530i	0.16984	−959.86897
TS24	737i	0.16862	−959.80297
TS25	531i	0.18015	−1073.19858
TS26	679i	0.17913	−1073.13590
TS27	524i	0.16914	−959.86733
TS28	730i	0.16819	−959.80396
TS29	666i	0.18020	−1073.18056
TS30	708i	0.17903	−1073.13580
TS31	542i	0.16935	−959.86845
TS32	737i	0.16798	−959.80211
TS33	1218i	0.15967	−847.08816
TS34	312i	0.16983	−922.32699
TS35	561i	0.14770	−845.94641
TS36	740i	0.14790	−845.91986
TS37	617i	0.14816	−845.95879
TS38	801i	0.14816	−845.94702
TS39	1185i	0.14353	−845.96901
TS40	651i	0.14805	−845.95239

Table S2. *Cont*.

Transition states	Imaginary frequencies	ZPE	Total energies
TS41	723i	0.14821	−845.94812
TS42	484i	0.14796	−845.95498
TS43	708i	0.14738	−845.91955
TS44	602i	0.14791	−845.95802
TS45	797i	0.14827	−845.94735
TS46	1183i	0.14377	−845.96996
TS47	604i	0.14797	−845.95805
TS48	780i	0.14849	−845.94994
TS49	1166i	0.14366	−845.96979
TS50	1320i	0.15951	−847.08665
TS51	383i	0.16955	−922.32717
TS52	452i	0.14813	−845.95624
TS53	752i	0.14732	−845.92060
TS54	603i	0.14829	−845.95893
TS55	784i	0.14858	−845.94859
TS56	673i	0.14811	−845.95308
TS57	706i	0.14858	−845.94705
TS58	1325i	0.15928	−847.08389
TS59	485i	0.16972	−922.32420
TS60	490i	0.14760	−845.94901
TS61	996i	0.16240	−847.08341
TS62	579i	0.17239	−922.29560
TS63	477i	0.15744	−386.27116
TS64	726i	0.15667	−386.23533
TS65	600i	0.15764	−386.27478
TS66	787i	0.15786	−386.26372
TS67	1178i	0.15330	−386.28669
TS68	1239i	0.15956	−847.08933
TS69	312i	0.16978	−922.32826
TS70	489i	0.14801	−845.95472
TS71	492i	0.14770	−845.95381
TS72	734i	0.14725	−845.91876
TS73	618i	0.14802	−845.95895
TS74	792i	0.14808	−845.94719
TS75	1190i	0.14372	−845.96969
TS76	1235i	0.15962	−847.08951
TS77	288i	0.16997	−922.32807
TS78	489i	0.14800	−845.95559
TS79	721i	0.14728	−845.91945
TS80	608i	0.14803	−845.95798
TS81	781i	0.14836	−845.94714
TS82	613i	0.14825	−845.95839
TS83	737i	0.14873	−845.94935

Table S3. CVT/SCT rate constants for the formation PCNs from cross–condensation of PhR with 2–CPR/3–CPR over the temperature range of 600–1200 *K* (units are s^{-1} and cm^3 $molecule^{-1}$ s^{-1} for unimolecular and bimolecular reactions, respectively).

**T(*K*)**	**CVT/SCT Rate Constants**			
	TS1	TS2	TS3	TS4
600	2.13×10^{-1}	8.14×10^{-6}	1.48×10^{-2}	4.78×10^{-4}
700	2.15×10^{1}	1.80×10^{-3}	2.24×10^{0}	6.85×10^{-2}
800	6.89×10^{2}	9.04×10^{-2}	9.76×10^{1}	2.86×10^{0}
900	1.03×10^{4}	1.89×10^{0}	1.85×10^{3}	5.23×10^{1}
1000	9.00×10^{4}	2.15×10^{1}	1.95×10^{4}	5.36×10^{2}
1100	5.32×10^{5}	1.57×10^{2}	1.35×10^{5}	3.61×10^{3}
1200	2.34×10^{6}	8.25×10^{2}	6.76×10^{5}	1.77×10^{4}
	TS5	TS6	TS7	TS8
600	2.19×10^{-9}	1.20×10^{-3}	1.16×10^{-1}	2.77×10^{-7}
700	1.70×10^{-6}	1.86×10^{-1}	1.24×10^{1}	9.88×10^{-5}
800	2.44×10^{-4}	6.32×10^{0}	4.18×10^{2}	8.15×10^{-3}
900	1.15×10^{-2}	1.22×10^{2}	6.45×10^{3}	2.53×10^{-1}
1000	2.46×10^{-1}	1.30×10^{3}	5.78×10^{4}	3.97×10^{0}
1100	3.00×10^{0}	9.13×10^{3}	3.48×10^{5}	3.78×10^{1}
1200	2.40×10^{1}	4.63×10^{4}	1.56×10^{6}	2.48×10^{2}
	TS9	TS10	TS11	TS12
600	2.80×10^{-1}	1.32×10^{-6}	1.06×10^{-2}	5.45×10^{-10}
700	2.58×10^{1}	4.07×10^{-4}	1.59×10^{0}	4.50×10^{-7}
800	7.75×10^{2}	3.02×10^{-2}	6.88×10^{1}	9.55×10^{-5}
900	1.10×10^{4}	8.61×10^{-1}	1.30×10^{3}	4.89×10^{-3}
1000	9.20×10^{4}	1.26×10^{1}	1.37×10^{4}	1.14×10^{-1}
1100	5.25×10^{5}	1.14×10^{2}	9.40×10^{4}	1.48×10^{0}
1200	2.24×10^{6}	7.15×10^{2}	4.70×10^{5}	1.26×10^{1}
	TS13	TS14	TS15	TS16
600	7.73×10^{-3}	3.94×10^{-10}	9.43×10^{-1}	1.37×10^{-6}
700	9.53×10^{-1}	3.61×10^{-7}	7.18×10^{1}	4.39×10^{-4}
800	4.67×10^{1}	6.02×10^{-5}	1.86×10^{3}	3.33×10^{-2}
900	9.68×10^{2}	3.21×10^{-3}	2.36×10^{4}	9.68×10^{-1}
1000	1.10×10^{4}	7.75×10^{-2}	1.80×10^{5}	1.44×10^{1}
1100	8.04×10^{4}	1.05×10^{0}	9.53×10^{5}	1.31×10^{2}
1200	4.23×10^{5}	9.16×10^{0}	3.83×10^{6}	8.29×10^{2}
	TS17	TS18	TS19	TS20
600	2.54×10^{-1}	1.44×10^{-6}	1.05×10^{0}	3.87×10^{-7}
700	2.49×10^{1}	3.60×10^{-4}	8.49×10^{1}	1.55×10^{-4}
800	7.80×10^{2}	2.85×10^{-2}	2.31×10^{3}	1.39×10^{-2}
900	1.14×10^{4}	8.61×10^{-1}	3.02×10^{4}	4.59×10^{-1}
1000	9.80×10^{4}	1.32×10^{1}	2.37×10^{5}	7.53×10^{0}
1100	5.70×10^{5}	1.23×10^{2}	1.28×10^{6}	7.42×10^{1}
1200	2.48×10^{6}	7.95×10^{2}	5.23×10^{6}	4.99×10^{2}

Table S3. *Cont*.

T(*K*)	CVT/SCT Rate Constants			
	TS21	TS22	TS23	TS24
600	4.25×10^{-4}	1.27×10^{-7}	7.26×10^{7}	3.08×10^{-7}
700	1.11×10^{-1}	5.67×10^{-5}	2.12×10^{8}	1.08×10^{-4}
800	7.31×10^{0}	5.48×10^{-3}	4.57×10^{8}	8.84×10^{-3}
900	1.91×10^{2}	1.91×10^{-1}	8.32×10^{8}	2.73×10^{-1}
1000	2.60×10^{3}	3.27×10^{0}	1.34×10^{9}	4.25×10^{0}
1100	2.21×10^{4}	3.34×10^{1}	1.99×10^{9}	4.03×10^{1}
1200	1.32×10^{5}	2.31×10^{2}	2.77×10^{9}	2.63×10^{2}
	TS25	TS26	TS27	TS28
600	1.01×10^{-2}	1.56×10^{-6}	5.42×10^{-2}	5.39×10^{-6}
700	1.06×10^{0}	4.34×10^{-4}	6.14×10^{0}	1.56×10^{-3}
800	3.51×10^{1}	2.97×10^{-2}	2.15×10^{2}	1.09×10^{-1}
900	5.34×10^{2}	7.97×10^{-1}	3.43×10^{3}	3.00×10^{0}
1000	4.71×10^{3}	1.11×10^{1}	3.15×10^{4}	4.25×10^{1}
1100	2.80×10^{4}	9.61×10^{1}	1.94×10^{5}	3.72×10^{2}
1200	1.24×10^{5}	5.81×10^{2}	8.81×10^{5}	2.28×10^{3}
	TS29	TS30	TS31	TS32
600	1.88×10^{-5}	7.24×10^{-6}	4.45×10^{-2}	6.18×10^{-7}
700	7.23×10^{-3}	8.30×10^{-4}	4.78×10^{0}	2.28×10^{-4}
800	6.35×10^{-1}	9.38×10^{-2}	1.60×10^{2}	1.92×10^{-2}
900	2.08×10^{1}	1.05×10^{0}	2.54×10^{3}	6.08×10^{-1}
1000	3.39×10^{2}	1.16×10^{1}	2.18×10^{4}	9.66×10^{0}
1100	3.35×10^{3}	1.27×10^{2}	1.30×10^{5}	9.29×10^{1}
1200	2.26×10^{4}	1.38×10^{2}	5.79×10^{5}	6.14×10^{2}
	TS33	TS35	TS36	TS37
600	9.67×10^{-13}	9.80×10^{-2}	1.25×10^{6}	1.67×10^{8}
700	1.73×10^{-12}	8.11×10^{0}	1.08×10^{7}	7.49×10^{8}
800	1.09×10^{-12}	2.21×10^{2}	5.51×10^{7}	2.30×10^{9}
900	1.60×10^{-12}	2.87×10^{3}	1.97×10^{8}	5.50×10^{9}
1000	2.20×10^{-12}	2.22×10^{4}	5.49×10^{8}	1.10×10^{10}
1100	2.94×10^{-12}	1.18×10^{5}	1.28×10^{9}	1.95×10^{10}
1200	3.81×10^{-12}	4.69×10^{5}	2.60×10^{9}	3.13×10^{10}
	TS38	TS39	TS40	TS41
600	7.29×10^{6}	1.27×10^{7}	7.26×10^{5}	1.66×10^{8}
700	5.16×10^{7}	9.36×10^{7}	6.07×10^{6}	8.77×10^{8}
800	1.87×10^{8}	4.24×10^{8}	2.87×10^{7}	3.06×10^{9}
900	5.98×10^{8}	1.39×10^{9}	9.59×10^{7}	8.11×10^{9}
1000	1.49×10^{9}	3.60×10^{9}	2.51×10^{8}	1.77×10^{10}
1100	3.17×10^{9}	7.90×10^{9}	5.52×10^{8}	3.35×10^{10}
1200	5.92×10^{9}	1.53×10^{10}	1.06×10^{9}	5.70×10^{10}

Table S3. *Cont.*

T(*K*)	CVT/SCT Rate Constants			
	TS42	TS43	TS44	TS45
600	1.45×10^{6}	7.42×10^{2}	9.93×10^{7}	1.11×10^{7}
700	9.68×10^{6}	2.24×10^{4}	4.07×10^{8}	7.16×10^{7}
800	3.98×10^{7}	2.90×10^{5}	1.17×10^{9}	2.90×10^{8}
900	1.19×10^{8}	2.13×10^{6}	2.67×10^{9}	8.61×10^{8}
1000	2.84×10^{8}	1.05×10^{7}	5.17×10^{9}	2.05×10^{9}
1100	5.76×10^{8}	3.90×10^{7}	8.86×10^{9}	4.19×10^{9}
1200	1.03×10^{9}	1.16×10^{8}	1.39×10^{10}	7.58×10^{9}
	TS46	TS47	TS48	TS49
600	6.86×10^{6}	1.82×10^{8}	2.28×10^{7}	6.26×10^{6}
700	4.96×10^{7}	7.05×10^{8}	1.40×10^{8}	5.21×10^{7}
800	2.19×10^{8}	1.94×10^{9}	5.47×10^{8}	2.55×10^{8}
900	6.98×10^{8}	4.28×10^{9}	1.58×10^{9}	8.77×10^{8}
1000	1.74×10^{9}	8.05×10^{9}	3.69×10^{9}	2.36×10^{9}
1100	3.71×10^{9}	1.35×10^{10}	7.39×10^{9}	5.29×10^{9}
1200	6.96×10^{9}	2.08×10^{10}	1.32×10^{10}	1.04×10^{10}
	TS50	TS52	TS53	TS54
600	2.84×10^{-13}	5.44×10^{8}	3.80×10^{6}	1.23×10^{8}
700	5.63×10^{-13}	1.82×10^{9}	3.03×10^{7}	4.81×10^{8}
800	9.85×10^{-13}	4.49×10^{9}	1.45×10^{8}	1.34×10^{9}
900	1.57×10^{-12}	9.07×10^{9}	4.97×10^{8}	2.97×10^{9}
1000	2.36×10^{-12}	1.59×10^{10}	1.34×10^{9}	5.61×10^{9}
1100	3.35×10^{-12}	2.51×10^{10}	3.04×10^{9}	9.47×10^{9}
1200	4.56×10^{-12}	3.68×10^{10}	6.04×10^{9}	1.47×10^{10}
	TS55	TS56	TS57	TS58
600	4.97×10^{6}	2.63×10^{6}	7.98×10^{7}	4.36×10^{-13}
700	3.69×10^{7}	1.64×10^{7}	4.33×10^{8}	5.70×10^{-13}
800	1.66×10^{8}	6.47×10^{7}	1.54×10^{9}	9.80×10^{-13}
900	5.37×10^{8}	1.88×10^{8}	4.14×10^{9}	1.54×10^{-12}
1000	1.37×10^{9}	4.43×10^{8}	9.12×10^{9}	1.62×10^{-12}
1100	2.96×10^{9}	8.93×10^{8}	1.74×10^{10}	2.23×10^{-12}
1200	5.62×10^{9}	1.60×10^{9}	2.99×10^{10}	2.97×10^{-12}
	TS60	TS61	TS62	TS63
600	8.43×10^{7}	8.16×10^{-14}	3.58×10^{-16}	1.50×10^{8}
700	2.88×10^{8}	2.00×10^{-13}	1.56×10^{-15}	5.19×10^{8}
800	7.18×10^{8}	4.04×10^{-13}	5.01×10^{-15}	1.31×10^{9}
900	1.46×10^{9}	7.12×10^{-13}	1.31×10^{-14}	2.70×10^{9}
1000	2.56×10^{9}	1.14×10^{-12}	2.94×10^{-14}	4.80×10^{9}
1100	4.04×10^{9}	1.70×10^{-12}	5.91×10^{-14}	7.66×10^{9}
1200	5.92×10^{9}	2.39×10^{-12}	1.09×10^{-13}	1.13×10^{10}

Table S3. *Cont*.

T(*K*)	CVT/SCT Rate Constants			
	TS64	TS65	TS66	TS67
600	1.10×10^{2}	3.97×10^{7}	1.03×10^{7}	1.24×10^{7}
700	3.10×10^{3}	1.73×10^{8}	7.07×10^{7}	9.03×10^{7}
800	3.79×10^{4}	5.19×10^{8}	2.48×10^{8}	4.05×10^{8}
900	2.67×10^{5}	1.22×10^{9}	7.26×10^{8}	1.32×10^{9}
1000	1.27×10^{6}	2.42×10^{9}	1.87×10^{9}	3.40×10^{9}
1100	4.57×10^{6}	4.22×10^{9}	3.90×10^{9}	7.41×10^{9}
1200	1.33×10^{7}	6.72×10^{9}	7.21×10^{9}	1.43×10^{10}
	TS68	TS69	TS70	TS71
600	3.31×10^{-13}	2.97×10^{-15}	2.99×10^{8}	2.45×10^{8}
700	6.80×10^{-13}	4.47×10^{-15}	9.96×10^{8}	8.25×10^{8}
800	1.17×10^{-12}	6.33×10^{-15}	2.45×10^{9}	2.05×10^{9}
900	1.78×10^{-12}	8.56×10^{-15}	4.95×10^{9}	4.17×10^{9}
1000	2.49×10^{-12}	1.12×10^{-14}	8.68×10^{9}	7.35×10^{9}
1100	3.28×10^{-12}	1.42×10^{-14}	1.37×10^{10}	1.17×10^{10}
1200	4.13×10^{-12}	1.77×10^{-14}	2.02×10^{10}	1.72×10^{10}
	TS72	TS73	TS74	TS75
600	2.10×10^{3}	4.58×10^{6}	2.53×10^{7}	7.62×10^{9}
700	5.52×10^{4}	3.08×10^{7}	1.74×10^{8}	2.16×10^{10}
800	6.44×10^{5}	1.29×10^{8}	7.40×10^{8}	4.73×10^{10}
900	4.35×10^{6}	3.92×10^{8}	2.28×10^{9}	8.69×10^{10}
1000	2.01×10^{7}	9.55×10^{8}	5.62×10^{9}	1.41×10^{11}
1100	7.03×10^{7}	1.98×10^{9}	1.17×10^{10}	2.11×10^{11}
1200	2.00×10^{8}	3.63×10^{9}	2.17×10^{10}	2.93×10^{11}
	TS76	TS78	TS79	TS80
600	1.30×10^{-15}	4.86×10^{7}	6.66×10^{2}	7.68×10^{7}
700	3.87×10^{-15}	1.95×10^{8}	1.89×10^{4}	3.28×10^{8}
800	8.81×10^{-15}	5.52×10^{8}	2.33×10^{5}	9.77×10^{8}
900	1.67×10^{-14}	1.24×10^{9}	1.64×10^{6}	2.28×10^{9}
1000	2.78×10^{-14}	2.37×10^{9}	7.84×10^{6}	4.49×10^{9}
1100	4.23×10^{-14}	4.03×10^{9}	2.81×10^{7}	7.82×10^{9}
1200	5.99×10^{-14}	6.26×10^{9}	8.16×10^{7}	1.24×10^{10}
	TS81	TS82	TS83	
600	1.56×10^{7}	1.05×10^{8}	2.04×10^{7}	
700	1.01×10^{8}	4.27×10^{8}	1.40×10^{8}	
800	4.09×10^{8}	1.23×10^{9}	5.93×10^{8}	
900	1.21×10^{9}	2.79×10^{9}	1.83×10^{9}	
1000	2.90×10^{9}	5.39×10^{9}	4.50×10^{9}	
1100	5.93×10^{9}	9.22×10^{9}	9.42×10^{9}	
1200	1.07×10^{10}	1.44×10^{10}	1.74×10^{10}	

Table S4. Cartesian coordinates for the transition states involved in PCN formation from cross-condenstaion of PhR with 2−CPR/3−CPR.

TS1

0 1

C	−0.924453	0.427435	0.000000
C	0.157965	−0.417999	−0.449104
C	−0.039508	−1.691006	−0.818853
C	−1.381003	−2.290513	−0.815213
C	−2.503498	−1.448686	−0.229442
C	−2.170422	−0.036584	0.081958
H	−3.865754	0.286905	−1.921187
C	−4.334484	−0.684342	−1.809014
C	−5.593144	−0.904839	−2.407591
C	−3.751982	−1.635612	−1.031112
C	−6.520752	−1.700628	−1.802414
C	−5.306174	−2.488880	0.213624
C	−6.412746	−2.370153	−0.563164
O	−4.738224	−2.939914	1.131813
H	−7.319095	−2.647767	−0.042128
H	−7.503164	−1.742986	−2.249871
H	−5.896563	−0.311657	−3.255474
H	−2.691498	−1.937249	0.741425
H	1.154434	−0.005844	−0.497063
Cl	1.240277	−2.675729	−1.389862
O	−1.597380	−3.401013	−1.231304
H	−2.975727	0.594922	0.428643
H	−0.693801	1.445260	0.272454
H	−3.949028	−2.671510	−1.294221

TS2

0 1

C	−1.365323	2.205423	0.394358
C	−2.558633	1.394530	0.323922
C	−2.520633	0.094101	−0.000580
C	−1.252958	−0.582942	−0.285913
C	−0.001808	0.280690	−0.305854
C	−0.165913	1.691372	0.125501
C	1.845650	0.050544	1.447136
C	3.095819	−0.595052	1.570915
C	1.137354	−0.457081	0.360052
C	3.521447	−1.178831	0.419484
C	3.029235	0.325015	−1.390017
C	2.664311	−0.900085	−0.749727

O	2.841611	1.469638	−1.268354
H	2.340706	−1.673571	−1.431697
H	4.468396	−1.684811	0.323525
O	−1.180613	−1.764301	−0.523501
H	0.730030	2.292197	0.167484
H	1.577936	0.945277	1.983658
H	−3.510978	1.849664	0.550116
H	−1.472208	3.241967	0.672411
H	3.699970	−0.576882	2.465373
H	0.239828	0.325166	−1.377101
Cl	−3.936045	−0.870334	−0.048195
H	0.979819	−1.532128	0.344722

TS3

0 1

C	−0.912830	2.173222	−0.158369
C	−2.048913	1.445886	−0.314333
C	−2.199658	0.041841	−0.178558
C	−1.232900	−0.899825	−0.061852
C	0.610678	0.316754	−0.385438
C	0.328013	1.545933	0.111166
C	2.550766	−0.114435	1.189139
C	3.845119	0.047153	0.894749
C	1.807490	−0.506295	−0.043347
C	4.047551	−0.185732	−0.530618
C	2.876249	−0.496497	−1.094851
H	4.997630	−0.117876	−1.034655
O	−0.843922	−2.001107	−0.062653
H	1.036170	2.032820	0.770406
H	2.089764	−0.021549	2.158476
H	−2.980390	1.973535	−0.454569
H	−0.995454	3.247833	−0.114025
H	4.628026	0.298675	1.591479
H	2.692634	−0.721059	−2.132150
H	0.104401	0.048514	−1.309972
Cl	−3.783142	−0.552731	0.257730
H	1.462619	−1.543654	0.078709

TS4

0 1

C	1.001249	−1.520904	−1.761126
C	2.015751	−1.009376	−0.960090
C	1.602284	−0.021496	0.011440
C	1.715299	−0.594301	1.241422

C	−0.181967	0.180968	−0.749813
C	−0.251388	−1.005548	−1.513786
C	−1.847265	−0.478841	1.047271
C	−3.164811	−0.567057	0.836764
C	−1.278827	0.601708	0.188463
C	−3.557509	0.414646	−0.167999
C	−2.477913	1.098694	−0.560848
H	−4.563794	0.558502	−0.525768
O	1.582036	−1.228251	2.192991
H	−1.164258	−1.566702	−1.638581
H	−1.260764	−1.076192	1.725210
H	3.026250	−1.384081	−0.985332
H	−2.435546	1.887318	−1.292782
H	0.266661	1.023726	−1.262174
H	1.184125	−2.367115	−2.406251
H	−3.841606	−1.248835	1.325172
Cl	2.092399	1.764407	0.060863
H	−0.887565	1.413507	0.811726

TS5

0 1

C	−0.787976	−1.128287	1.790816
C	−1.766365	−1.195456	0.849916
C	−2.148776	−0.160260	−0.037223
C	−1.548680	1.051438	−0.193248
C	0.336487	0.863761	0.899849
C	−0.081033	0.087427	1.942225
H	−0.056886	−1.356309	−1.038780
C	0.930856	−1.019307	−0.771976
C	1.973318	−1.838623	−0.903958
C	1.079424	0.399125	−0.347198
C	3.315751	−1.400528	−0.593849
C	2.530749	0.849349	−0.108213
C	3.588259	−0.142942	−0.222778
O	2.760429	2.004078	0.171202
H	4.587433	0.193549	0.003614
H	4.119440	−2.117850	−0.677568
H	1.828568	−2.848281	−1.255097
H	0.619552	1.882542	1.128771
H	−2.401349	−2.068727	0.822608
Cl	−3.750988	−0.237717	−0.724778
O	−1.445087	2.061979	−0.770787
H	−0.002603	0.500019	2.942724
H	−0.737654	−1.895913	2.546686

H	0.755474	1.029525	−1.181236

TS6

0 1

C	2.970056	−1.392586	−0.498929
C	3.815810	−0.262926	−0.177044
C	3.319645	0.932334	0.171957
C	1.883425	1.147491	0.268661
C	0.971258	0.020634	−0.209695
C	1.642550	−1.276344	−0.494194
C	−0.580528	−1.178497	1.431107
C	−1.939961	−1.153991	1.800767
C	−0.240818	−0.065161	0.672682
C	−2.723544	−0.383240	0.994108
C	−2.000370	−0.884425	−1.238041
C	−2.041302	0.102952	−0.199299
O	−1.374401	−1.848414	−1.456295
H	−3.789014	−0.274766	1.120140
O	1.408885	2.181155	0.683128
H	1.004595	−2.111582	−0.741351
H	0.065254	−2.028465	1.578475
H	4.886645	−0.404155	−0.207610
H	3.436194	−2.334200	−0.743448
H	−2.356044	−1.751605	2.597713
H	3.950698	1.763081	0.445164
H	−0.521835	0.906504	1.068111
Cl	−2.236645	1.737270	−0.758029
H	0.613286	0.402224	−1.177838

TS7

0 1

C	−2.350546	1.089168	−1.423752
C	−3.293756	0.278857	−0.863675
C	−3.177603	−0.507972	0.304095
C	−2.054254	−0.740927	1.030763
C	−0.500131	0.167503	−0.175017
C	−1.069265	1.206269	−0.844519
C	1.216492	1.605338	1.026362
C	2.475107	1.812577	0.624909
C	0.771886	0.244405	0.605084
C	2.959490	0.649887	−0.107805
C	1.978928	−0.255140	−0.130442
H	3.936032	0.542106	−0.547119
O	−1.484435	−1.309406	1.880596

H	−0.564768	2.165035	−0.869953
H	0.598317	2.277603	1.597076
H	−4.290413	0.319303	−1.278774
H	−2.654293	1.773202	−2.200164
H	3.065226	2.693996	0.814853
H	−4.079327	−0.797001	0.827085
H	0.646035	−0.377003	1.502425
Cl	2.014211	−1.799669	−0.861034
H	−0.761004	−0.827403	−0.528639

TS8

0 1

C	2.347213	−0.763211	1.544614
C	2.831848	−1.209849	0.353560
C	2.056583	−0.761250	−0.820875
C	2.510398	0.516937	−1.274089
C	0.468239	−0.429995	0.249112
C	1.117537	−0.081297	1.431822
C	−0.720644	1.828297	0.078333
C	−1.965087	2.076133	0.499110
C	−0.619212	0.406604	−0.371081
C	−2.775168	0.868311	0.395441
C	−2.002110	−0.101321	−0.095080
H	−3.813535	0.779066	0.664055
O	2.390105	1.644808	−1.009623
H	0.831386	0.757406	2.044887
H	0.109207	2.511398	0.035666
H	3.779432	−1.713564	0.250890
H	2.902770	−0.864307	2.464836
H	−2.336300	3.020777	0.860778
H	1.758491	−1.429636	−1.615660
H	−0.480070	0.395433	−1.458523
Cl	−2.451881	−1.718529	−0.424553
H	0.312180	−1.496204	0.102264

TS9

0 1

C	2.066924	−0.663023	1.802231
C	3.104820	0.226846	1.325293
C	2.956999	0.964584	0.219186
C	1.700518	0.954125	−0.517705
C	0.685422	−0.161646	−0.184185
C	0.944511	−0.854509	1.111890
H	−0.394650	2.172412	0.694517

C	−1.120895	1.528513	0.212674
C	−2.469894	1.929121	0.203480
C	−0.729190	0.332291	−0.307097
C	−3.476682	1.008713	0.237736
C	−2.239526	−1.151076	0.239393
C	−3.367176	−0.394854	0.309436
O	−1.722648	−2.197253	0.172115
H	−4.212748	−0.961557	0.675806
H	−4.485052	1.383324	0.337128
H	−2.717147	2.969978	0.336373
H	4.020804	0.292791	1.893998
O	1.441553	1.770307	−1.366162
H	0.188336	−1.548396	1.441257
H	2.226671	−1.192441	2.728399
H	−1.282885	−0.030865	−1.166897
H	3.712363	1.650025	−0.130027
Cl	0.991652	−1.390248	−1.480716

TS10
0 1

C	−2.091199	0.447975	1.815807
C	−3.164181	−0.262027	1.148136
C	−3.028276	−0.768751	−0.082901
C	−1.774531	−0.642613	−0.808710
C	−0.632282	0.150931	−0.147760
C	−0.914909	0.645848	1.225055
C	1.048342	−1.481336	0.823665
C	2.386144	−1.877565	0.618169
C	0.614509	−0.696271	−0.247397
C	3.055590	−1.088600	−0.265593
C	2.541865	1.200218	0.127213
C	2.288858	0.088279	−0.732641
O	2.263791	1.631405	1.168538
H	2.171714	0.355095	−1.770930
H	4.104084	−1.205776	−0.486146
O	−1.597397	−1.139329	−1.894659
H	−0.117068	1.182254	1.713730
H	0.534027	−1.564835	1.766828
H	−4.101009	−0.378177	1.673080
H	−2.265463	0.825939	2.811055
H	2.873881	−2.671791	1.162706
H	0.667656	−1.162446	−1.228343
Cl	−0.432840	1.628616	−1.181500
H	−3.820257	−1.299866	−0.585779

TS11

0 1

C	−1.674341	1.870141	−0.477678
C	−2.736854	1.165024	0.036479
C	−2.685393	−0.018761	0.779901
C	−1.568402	−0.770864	1.033363
C	−0.055839	0.057604	−0.276419
C	−0.375940	1.396338	−0.284418
C	1.899043	0.316280	1.287625
C	3.137826	0.680267	0.939892
C	1.280153	−0.452926	0.168047
C	3.411217	0.224750	−0.419713
C	2.341215	−0.423505	−0.890126
H	4.335116	0.392515	−0.948480
O	−1.093051	−1.729183	1.502551
H	0.411173	2.087381	−0.002046
H	1.406687	0.492330	2.229218
H	−3.704931	1.643526	0.006662
H	−1.837529	2.892386	−0.779164
H	3.839266	1.214303	1.560054
H	2.218287	−0.871232	−1.861258
H	1.135604	−1.488040	0.500716
H	−3.508453	−0.262049	1.437540
Cl	−0.787466	−0.972573	−1.502669

TS12

0 1

C	1.691150	−0.378926	1.723635
C	2.503874	−0.094289	0.653318
C	1.763260	0.104497	−0.624957
C	1.875791	1.476563	−0.758540
C	0.106590	−0.550272	0.043592
C	0.337104	−0.459994	1.428874
C	−1.459045	1.431933	−0.171687
C	−2.697436	1.483217	0.327691
C	−1.146469	0.025019	−0.571603
C	−3.272636	0.142361	0.332825
C	−2.384633	−0.719724	−0.170495
H	−4.259438	−0.101260	0.690436
O	1.810855	2.613452	−0.618717
H	−0.450787	−0.154881	2.098903
H	−0.785591	2.262229	−0.293604
H	3.560312	0.097736	0.730321

H	−2.495311	−1.782906	−0.292252
H	2.087838	−0.419174	2.727166
H	−3.202753	2.369464	0.675102
H	−1.048728	−0.032220	−1.660291
Cl	0.374599	−2.200912	−0.675087
H	1.834130	−0.493414	−1.525484

TS13
0 1

C	−1.056268	−1.473642	1.506950
C	−2.031325	−1.818316	0.611567
C	−2.610143	−1.010958	−0.383700
C	−2.183288	0.213683	−0.789449
C	−0.365127	0.620160	0.438824
C	−0.567432	−0.157712	1.553020
H	−0.362147	−1.511844	−1.801107
C	0.451672	−1.212516	−1.160074
C	1.349457	−2.106047	−0.748820
C	0.528867	0.220613	−0.751257
C	2.445077	−1.727956	0.124221
C	1.948720	0.594412	−0.315573
C	2.718067	−0.443426	0.376228
O	2.400635	1.687932	−0.538676
H	3.565622	−0.125757	0.962297
H	3.066366	−2.507694	0.540112
H	1.267711	−3.137165	−1.055944
H	−2.511646	−2.775788	0.754762
O	−2.191786	1.098850	−1.547624
H	−0.495667	0.319155	2.524120
H	−0.883444	−2.129313	2.346073
H	0.268672	0.877559	−1.579090
Cl	−0.339092	2.339298	0.705363
H	−3.591375	−1.261233	−0.763612

TS14
0 1

C	−1.565616	2.251411	0.013457
C	−2.637221	1.605968	0.749605
C	−2.898336	0.300906	0.611050
C	−2.140189	−0.500593	−0.351648
C	−0.792386	0.052663	−0.802653
C	−0.716331	1.539879	−0.725423
C	0.408543	−0.232114	1.431967
C	1.720004	−0.229994	1.867065

C	0.309148	−0.570868	0.060858
C	2.667615	−0.245564	0.866013
C	2.476404	1.038430	−0.816484
C	2.088659	−0.246922	−0.510424
O	2.690300	2.163234	−0.910501
H	3.726887	−0.152873	1.034842
O	−2.561570	−1.544518	−0.784851
H	0.071228	2.035648	−1.272260
H	−0.398500	0.256780	1.951917
H	−3.250232	2.213217	1.399571
H	−1.469617	3.324215	0.074936
H	2.006423	−0.104837	2.900451
H	−3.724832	−0.184236	1.105516
H	−0.642202	−0.296120	−1.822521
H	2.155172	−1.016746	−1.270699
Cl	0.410239	−2.329647	−0.288190

TS15

0 1

C	2.337928	−1.705684	−0.539753
C	3.226783	−0.747235	−0.143379
C	2.945692	0.512299	0.419348
C	1.726264	1.099052	0.576521
C	0.413010	−0.243640	−0.499246
C	0.951314	−1.494309	−0.431810
C	−1.372641	0.199444	1.228527
C	−2.266625	−0.721130	1.582647
C	−1.046185	0.035842	−0.232140
C	−2.632885	−1.522283	0.408543
C	−1.954189	−1.086704	−0.650267
H	−3.354169	−2.322767	0.413381
O	1.088413	2.034215	0.867120
H	0.305037	−2.339217	−0.223447
H	−0.911515	0.956322	1.836663
H	4.273983	−1.013113	−0.145929
H	2.711810	−2.689893	−0.772516
H	−2.675341	−0.860369	2.569569
H	3.713494	1.001387	1.003770
H	0.911334	0.468033	−1.151392
Cl	−1.509211	1.530334	−1.124492
H	−1.996164	−1.443696	−1.664641

TS16

0 1

C	2.212068	−1.830653	−0.266966
C	2.826634	−0.661785	−0.607911
C	1.949160	0.529187	−0.551247
C	2.071248	1.166617	0.722477
C	0.359598	−0.524829	−0.640650
C	0.830213	−1.699108	−0.042573
C	−1.323606	0.006146	1.199294
C	−2.449596	−0.692771	1.329372
C	−0.965273	0.088689	−0.253487
C	−2.932468	−1.095827	0.003169
C	−2.098119	−0.634323	−0.925502
H	−3.824708	−1.672151	−0.175876
O	1.743896	1.112509	1.832513
H	0.280518	−2.257698	0.697260
H	−0.722224	0.445250	1.974217
H	3.891027	−0.573937	−0.752847
H	2.768018	−2.742082	−0.105421
H	−2.944279	−0.926934	2.257352
H	1.839011	1.230518	−1.363393
H	0.521530	−0.464485	−1.714563
H	−2.158031	−0.748444	−1.993813
Cl	−0.969481	1.817755	−0.781111

TS17

0 1

C	−2.578685	1.044787	0.420008
C	−3.303215	−0.176297	0.697920
C	−2.838940	−1.390519	0.375120
C	−1.550217	−1.539333	−0.286260
C	−0.694492	−0.291687	−0.483089
C	−1.369576	0.986374	−0.135303
H	0.237500	0.723077	1.947276
C	0.933299	0.062065	1.442916
C	2.225618	−0.103760	1.983074
C	0.595075	−0.511001	0.257484
C	3.305344	−0.254400	1.163074
C	2.269724	−0.250139	−1.094339
C	3.332309	−0.249652	−0.248949
O	1.807105	−0.335756	−2.164213
H	4.259986	−0.005466	−0.748729
H	4.280597	−0.260354	1.627948
H	2.395973	0.068837	3.033710
H	−4.267459	−0.082080	1.175447

O	−1.124785	−2.610427	−0.654186
H	−3.032950	1.995019	0.648426
H	1.058276	−1.465138	0.020383
H	−3.395203	−2.293113	0.570228
H	−0.442386	−0.265968	−1.549393
Cl	−0.500609	2.423296	−0.513414

TS18

0 1

C	−2.792950	0.035807	−0.544725
C	−2.686347	1.429284	−0.906891
C	−1.660754	2.204557	−0.524633
C	−0.587511	1.649130	0.281538
C	−0.506939	0.135326	0.413325
C	−1.796229	−0.555145	0.115929
C	1.137739	−1.620331	−0.476831
C	2.405804	−1.610246	−1.098070
C	0.562478	−0.350272	−0.559469
C	2.959168	−0.371848	−1.212106
C	2.538001	0.807104	0.816432
C	2.189595	0.706947	−0.549766
O	2.432416	0.298268	1.850672
H	1.929266	1.643933	−1.014401
H	3.953850	−0.203781	−1.592109
O	0.268048	2.341913	0.789406
H	0.819530	−2.386916	0.208526
H	−3.498766	1.859193	−1.474194
H	−3.694859	−0.505772	−0.777371
H	2.945403	−2.502445	−1.379954
H	0.427739	0.035996	−1.567667
H	−1.620059	3.259758	−0.740895
H	−0.172386	−0.095722	1.423623
Cl	−1.956809	−2.175739	0.662672

TS19

0 1

C	1.989954	1.499518	0.331342
C	3.015677	0.627998	0.134552
C	2.947463	−0.751281	−0.164785
C	1.837551	−1.530468	−0.177198
C	0.297013	−0.214273	0.619571
C	0.652109	1.045136	0.247063
C	−1.611153	−0.898456	−0.946006
C	−2.901137	−0.552453	−0.881943

C	−1.027211	−0.882292	0.430176
C	−3.240140	−0.215357	0.493720
C	−2.159626	−0.369417	1.266715
H	−4.214512	0.104406	0.825196
O	1.315530	−2.573662	−0.250303
H	−1.057612	−1.207942	−1.816325
H	4.012271	1.044553	0.112548
H	2.189501	2.556389	0.391702
H	−3.591782	−0.523379	−1.708538
H	−2.086845	−0.198668	2.327430
H	−0.880601	−1.935300	0.700461
H	3.803578	−1.231314	−0.618189
Cl	−0.494962	2.119405	−0.456370
H	0.913123	−0.601270	1.427182

TS20

0 1

C	1.949605	1.534731	0.235622
C	2.717110	0.473481	0.603160
C	1.976439	−0.792219	0.786428
C	2.203248	−1.698964	−0.329077
C	0.297899	0.053118	0.918187
C	0.578951	1.230930	0.205905
C	−1.377131	−1.151578	−0.646859
C	−2.683684	−0.895615	−0.771505
C	−0.936016	−0.786509	0.734527
C	−3.175859	−0.284754	0.454186
C	−2.172503	−0.188025	1.332919
H	−4.191720	0.036378	0.616168
O	1.799685	−2.002651	−1.372119
H	−0.729911	−1.589740	−1.386187
H	3.792564	0.517443	0.663893
H	−2.214343	0.221371	2.328264
H	2.359752	2.483897	−0.071415
H	−3.286085	−1.093640	−1.642654
H	−0.751878	−1.729821	1.265159
H	2.035107	−1.343878	1.715459
Cl	−0.484366	1.964323	−0.918951
H	0.554898	0.158144	1.969468

TS21

0 1

C	1.565102	0.006096	−1.545675
C	2.051572	−1.230761	−1.246905

C	2.109209	−1.842089	0.022065
C	1.604686	−1.339284	1.181633
C	0.416484	0.366892	0.605978
C	1.134166	0.812207	−0.467527
H	−0.328692	−2.016970	−0.934218
C	−1.086446	−1.290792	−0.689624
C	−2.205688	−1.221539	−1.409134
C	−0.864451	−0.451832	0.519526
C	−3.248163	−0.273444	−1.085147
C	−1.998768	0.534943	0.839620
C	−3.162764	0.550732	−0.032654
O	−1.909535	1.259915	1.804137
H	−3.932418	1.265982	0.210011
H	−4.121637	−0.237952	−1.720171
H	−2.351062	−1.879769	−2.251306
H	2.527793	−1.781157	−2.045975
O	1.378050	−1.473573	2.319888
H	1.748852	0.441031	−2.513670
H	−0.879000	−1.126678	1.382941
H	2.765068	−2.686818	0.176075
Cl	1.796178	2.411886	−0.407585
H	0.398316	1.002760	1.480127

TS22

0 1

C	−2.272741	−0.615087	−1.576693
C	−3.371040	−0.336480	−0.680356
C	−3.201375	−0.137384	0.635726
C	−1.878181	−0.192476	1.235046
C	−0.729099	−0.650064	0.342226
C	−1.026728	−0.715156	−1.115166
C	1.062044	1.191604	0.149183
C	2.439713	1.334409	0.395179
C	0.549300	0.033186	0.748072
C	3.025637	0.176986	0.797092
C	2.377482	−1.891639	−0.307073
C	2.132719	−1.000203	0.809837
O	2.027436	−2.126099	−1.389062
H	4.078274	0.093724	1.014153
O	−1.678403	0.054261	2.404995
H	−0.200388	−0.908647	−1.782431
H	−4.363938	−0.265564	−1.101020
H	−2.476234	−0.716376	−2.630918
H	2.988287	2.245521	0.216478

H	−4.020063	0.110998	1.292252
H	−0.611501	−1.699402	0.651076
H	1.997933	−1.581734	1.712896
H	0.632894	0.056637	1.830347
Cl	0.266829	2.120786	−1.054630

TS23

0 1

C	−2.118433	0.002375	−1.847541
C	−3.156895	0.020259	−0.963580
C	−3.094241	−0.000708	0.447937
C	−1.994206	−0.198104	1.218709
C	−0.467324	−0.701497	−0.232701
C	−0.785469	−0.055311	−1.387070
C	1.771948	0.449451	0.059678
C	2.952953	0.005252	−0.377782
C	0.875535	−0.687000	0.424102
C	2.919795	−1.451050	−0.362083
C	1.733189	−1.869344	0.089807
H	3.740232	−2.077518	−0.670677
O	−1.495266	−0.396675	2.259018
H	−0.021584	0.497880	−1.921009
H	−4.145426	0.189331	−1.365504
H	−2.320140	0.224804	−2.883315
H	3.788851	0.613914	−0.676865
H	−3.933643	0.385731	1.010293
H	−1.088325	−1.557529	0.017891
H	1.404405	−2.887573	0.209659
H	0.713296	−0.660482	1.510018
Cl	1.294315	2.079936	0.251795

TS24

0 1

C	2.346909	−0.763657	1.544664
C	2.831826	−1.209874	0.353559
C	2.056695	−0.761106	−0.820899
C	2.510568	0.517122	−1.273930
C	0.468174	−0.430140	0.248888
C	1.117205	−0.081824	1.431852
C	−0.720470	1.828267	0.078441
C	−1.964856	2.076143	0.499362
C	−0.619188	0.406635	−0.371210
C	−2.775042	0.868401	0.395593
C	−2.002117	−0.101205	−0.095185

H	−3.813374	0.779180	0.664349
O	2.390297	1.644978	−1.009406
H	0.830846	0.756607	2.045194
H	0.109355	2.511385	0.035608
H	3.779557	−1.713321	0.250929
H	2.902366	−0.864878	2.464934
H	−2.335969	3.020780	0.861153
H	1.758694	−1.429403	−1.615792
H	0.312165	−1.496301	0.101654
Cl	−2.452009	−1.718355	−0.424761
H	−0.480077	0.395584	−1.458655

TS25

0 1

C	−2.037953	−0.916806	1.000429
C	−2.814004	−0.108282	0.081409
C	−2.279438	0.830447	−0.709354
C	−0.843031	1.068375	−0.693113
C	−0.009399	0.331143	0.343110
C	−0.725040	−0.723250	1.103233
H	0.957030	−2.200605	−0.282673
C	1.651904	−1.394642	−0.491382
C	2.956042	−1.716385	−0.923502
C	1.289156	−0.105598	−0.254679
C	4.021267	−0.952106	−0.547895
C	2.937417	0.844723	0.777531
C	4.019175	0.184612	0.291183
O	2.453549	1.746240	1.345242
H	4.936485	0.461682	0.792953
H	5.005081	−1.308085	−0.817171
H	3.144078	−2.661230	−1.407934
H	0.241507	1.117999	1.073215
O	−0.316729	1.840930	−1.462129
H	−0.142089	−1.317089	1.792839
H	−2.552154	−1.660697	1.586541
H	1.758359	0.656927	−0.871035
Cl	−4.504105	−0.425803	0.036184
H	−2.869657	1.399015	−1.408583

TS26

0 1

C	2.172087	1.301368	0.476429
C	2.729363	0.061385	−0.019278
C	1.995483	−1.033213	−0.262677

C	0.560749	−1.011272	−0.040421
C	−0.071839	0.247998	0.513947
C	0.867029	1.384185	0.725758
C	−2.123285	1.695209	0.157162
C	−3.384111	1.571253	−0.468072
C	−1.252108	0.708968	−0.319188
C	−3.611524	0.352499	−1.027218
C	−2.732881	−1.379004	0.395253
C	−2.537499	−0.641361	−0.789845
O	−2.665511	−1.386107	1.549828
H	−2.060277	−1.211570	−1.569351
H	−4.545084	0.074802	−1.488639
O	−0.128089	−1.984271	−0.277684
H	0.442678	2.305607	1.094181
H	−1.988281	2.213424	1.094135
H	2.835904	2.135125	0.634760
H	−4.148530	2.333986	−0.440353
H	−0.473134	−0.038040	1.492978
H	−1.040721	0.746571	−1.385509
Cl	4.425308	0.047130	−0.295482
H	2.427226	−1.946190	−0.637258

TS27

0 1

C	−1.429389	−1.349531	0.001582
C	−2.291255	−0.296808	0.040158
C	−2.003635	1.080241	−0.116841
C	−0.768755	1.636578	−0.174649
C	0.559926	0.000051	0.312538
C	−0.047608	−1.119441	−0.165587
C	2.639282	−0.263816	−1.122525
C	3.803585	−0.793204	−0.730895
C	1.976055	0.390451	0.042377
C	3.977368	−0.569696	0.699616
C	2.921314	0.102507	1.168738
H	4.828908	−0.897261	1.273114
O	−0.081392	2.583198	−0.197419
H	0.528698	−1.828553	−0.747883
H	2.235129	−0.254952	−2.121265
H	−1.834500	−2.344893	−0.074911
H	4.516324	−1.297055	−1.363221
H	2.749663	0.417311	2.184481
H	0.123523	0.430936	1.210533
H	1.984575	1.476651	−0.130743

H	−2.796941	1.737240	−0.443445
Cl	−3.982681	−0.647588	0.121507

TS28
0 1

C	1.375733	−0.984580	1.136758
C	2.011097	−0.470718	0.044009
C	1.171512	0.297204	−0.884980
C	1.151934	1.671786	−0.482951
C	−0.424455	−0.623217	−0.261143
C	−0.016102	−0.841886	1.058833
C	−2.385835	0.890795	0.370042
C	−3.629309	0.474041	0.629862
C	−1.756131	−0.026357	−0.626118
C	−3.900252	−0.742343	−0.128634
C	−2.821413	−1.058799	−0.850679
H	−4.828229	−1.289530	−0.100496
O	0.639230	2.392458	0.268454
H	−0.656407	−0.692393	1.912784
H	−1.887024	1.751826	0.778581
H	−2.703040	−1.903666	−1.508320
H	−0.100681	−1.386663	−0.963989
H	1.917065	−1.379683	1.981990
H	−4.332263	0.952340	1.292126
H	−1.630258	0.523642	−1.567190
Cl	3.720031	−0.434129	−0.120512
H	1.205783	0.149089	−1.954423

TS29
0 1

C	−1.452108	−0.414717	1.367304
C	−2.175117	−0.186630	0.236381
C	−2.011706	0.867420	−0.691325
C	−1.027654	1.807046	−0.637673
C	0.404040	1.076075	0.760180
C	−0.432431	0.506972	1.682337
H	−0.308246	−1.081969	−1.112878
C	0.702601	−1.005510	−0.748393
C	1.498468	−2.074620	−0.770470
C	1.182967	0.338388	−0.324287
C	2.871085	−1.996947	−0.325741
C	2.649237	0.381643	0.137792
C	3.421346	−0.850231	0.093944
O	3.132026	1.425566	0.514444

H	4.442545	−0.786052	0.434333
H	3.464429	−2.899884	−0.339182
H	1.124594	−3.020194	−1.130699
H	0.959522	1.948154	1.076522
O	−0.522040	2.740441	−1.129465
H	−0.409306	0.892799	2.696276
H	−1.840906	−1.095751	2.106168
H	1.198636	0.967502	−1.221835
H	−2.834220	1.122997	−1.342466
Cl	−3.556547	−1.180529	−0.054492

TS30
0 1

C	3.618577	1.256552	0.009803
C	4.180793	−0.055919	−0.219024
C	3.415699	−1.148099	−0.364518
C	1.964944	−1.038208	−0.346238
C	1.372309	0.250840	0.181652
C	2.304632	1.414148	0.171888
C	−0.682260	1.723824	−0.165561
C	−2.059344	1.540427	−0.391706
C	0.023431	0.545459	−0.425368
C	−2.420173	0.229915	−0.378951
C	−1.350383	−0.893109	1.431220
C	−1.372793	−0.723216	0.022382
O	−0.928853	−0.431410	2.409766
O	1.245131	−1.946053	−0.708957
H	1.887734	2.391977	0.354675
H	−0.294781	2.564786	0.385645
H	5.254700	−0.143845	−0.298946
H	4.281508	2.106459	0.048153
H	−2.783650	2.333625	−0.491935
H	3.828886	−2.118308	−0.589740
H	−0.095228	0.130411	−1.422571
H	1.206645	0.036097	1.248691
Cl	−4.038813	−0.310821	−0.556373
H	−1.128803	−1.613224	−0.535576

TS31
0 1

C	2.554265	−1.895246	−0.138903
C	3.626012	−1.078158	−0.351186
C	3.673648	0.329960	−0.252293
C	2.626799	1.177058	−0.070877

C	0.909289	−0.126969	−0.227578
C	1.299260	−1.355712	0.210869
C	−0.903631	0.166667	1.520056
C	−2.198193	−0.122833	1.370290
C	−0.326042	0.592356	0.211174
C	−2.541895	0.056778	−0.034121
C	−1.480875	0.460052	−0.733868
O	2.185562	2.261468	−0.039461
H	0.667061	−1.900321	0.902359
H	−0.338870	0.140907	2.437051
H	4.586929	−1.549987	−0.497077
H	2.724081	−2.957231	−0.059327
H	−2.897899	−0.426479	2.130133
H	4.630073	0.806476	−0.083428
H	−0.069029	1.658611	0.287926
H	1.290781	0.166570	−1.202841
Cl	−4.121923	−0.230671	−0.637187
H	−1.436751	0.667405	−1.788307

TS32

0 1

C	2.629249	−1.461185	1.020624
C	3.344351	−1.121263	−0.087876
C	2.655346	−0.166927	−0.980240
C	2.927220	1.178607	−0.572630
C	0.909132	−0.641932	−0.278919
C	1.313707	−0.956641	1.018625
C	−0.641172	1.246598	0.471969
C	−1.942712	1.123482	0.739915
C	−0.251250	0.267311	−0.588564
C	−2.477313	0.049723	−0.086684
C	−1.524627	−0.476366	−0.856725
O	2.589492	1.950221	0.230346
H	0.781168	−0.645207	1.902154
H	0.049649	1.940561	0.916594
H	4.379681	−1.386448	−0.228695
H	3.069895	−2.002244	1.844493
H	−2.528085	1.699007	1.436340
H	2.599256	−0.298528	−2.050963
H	−0.001592	0.835742	−1.493402
H	1.002261	−1.457418	−0.992705
Cl	−4.123075	−0.426511	−0.024413
H	−1.627478	−1.284997	−1.558522

TS33

0 2

C	2.457152	−1.266512	0.368763
C	2.288460	0.153058	0.645876
C	1.213981	0.580102	−0.022647
C	0.595340	−0.536627	−0.806711
C	1.493905	−1.680916	−0.460044
C	−1.912726	0.122476	−0.970754
C	−2.811187	0.307385	0.006264
C	−0.865380	−0.801290	−0.480167
C	−2.381315	−0.418156	1.194811
C	−1.229280	−1.050938	0.932518
H	−2.915645	−0.432703	2.130397
H	1.346051	−2.678198	−0.839306
H	−1.940888	0.525875	−1.968695
H	2.922239	0.752922	1.275809
H	−0.648497	−1.664935	1.599466
H	0.678266	−0.312405	−1.874262
H	3.248530	−1.872492	0.778131
H	−3.710016	0.897714	−0.060885
Cl	0.603028	2.174700	−0.048492
H	−1.104163	−1.832702	−1.047435
H	−1.221051	−2.804465	−1.616305

TS34

0 2

C	−1.795762	2.057834	0.560506
C	−0.667926	2.051720	−0.156787
C	−0.402981	0.672406	−0.670099
C	−1.563057	−0.090571	−0.107220
C	−2.364569	0.717258	0.592333
H	−2.235680	2.912334	1.047722
H	−0.001443	2.875457	−0.348679
H	−3.276245	0.429569	1.087033
C	2.007283	−1.874270	0.078640
C	1.826378	−1.228356	1.374521
C	1.199446	−0.058839	1.198620
C	0.965160	0.143394	−0.258132
C	1.480205	−1.098927	−0.878730
H	2.484696	−2.828988	−0.068811
H	2.146853	−1.643740	2.315784
H	1.698064	0.967047	−0.600156
H	1.454232	−1.296211	−1.936770
O	2.588252	2.180132	−0.682256

H	3.141768	1.956786	0.077435
H	−0.463512	0.666453	−1.761805
H	0.900996	0.650257	1.952332
Cl	−1.803576	−1.757642	−0.394585

TS35
0 2

C	1.729304	−0.453604	−0.945711
C	1.018257	0.388930	−0.088351
C	0.647678	−0.367352	1.118235
C	1.293503	−1.677869	0.923738
C	1.886038	−1.702779	−0.300759
H	2.080794	−0.181428	−1.924819
H	1.195805	−2.500214	1.610939
H	2.375185	−2.556798	−0.739350
C	−2.553676	−0.808318	−0.732416
C	−2.806103	0.390660	0.039968
C	−1.672751	0.729272	0.712938
C	−0.680296	−0.250673	0.403694
C	−1.279340	−1.199105	−0.521471
H	−3.268649	−1.294713	−1.373534
H	−3.743189	0.920765	0.072279
H	−0.774172	−2.042048	−0.956083
H	0.669465	0.116050	2.084885
H	−1.534776	1.566867	1.373494
Cl	1.029639	2.098621	−0.223941

TS36
0 2

C	−1.328522	−1.705632	0.951974
C	−0.358740	−0.775786	1.246896
C	−0.763785	0.431772	−0.214019
C	−1.716224	−0.406883	−0.922648
C	−1.945923	−1.600471	−0.337780
H	−1.737643	−2.338503	1.724610
H	−0.103785	−0.524650	2.264300
H	−2.168758	−0.062103	−1.838571
H	−2.595208	−2.355127	−0.749564
C	2.768403	0.340534	0.167286
C	2.542294	−0.779289	−0.743972
C	1.239974	−1.072216	−0.768486
C	0.527915	−0.149999	0.165091
C	1.602894	0.722641	0.696726
H	3.729915	0.781377	0.372120

H	3.317110	−1.283898	−1.297376
H	1.431895	1.520436	1.398522
H	0.743447	−1.843622	−1.329697
Cl	−1.060393	2.122238	−0.117103

TS37

0 2

C	1.765221	1.882681	0.053919
C	2.013871	0.487269	0.193258
C	1.027502	−0.411958	0.022143
C	−0.536181	1.460844	−0.527176
C	0.537179	2.333532	−0.373069
H	2.582290	2.572247	0.190850
H	3.015720	0.142386	0.394495
H	0.404547	3.370977	−0.639964
C	−2.572534	−0.716075	−0.308762
C	−2.472579	0.116004	0.840133
C	−1.160300	0.534328	0.975992
C	−0.380898	−0.009795	−0.172991
C	−1.360425	−0.839099	−0.904832
H	−3.487808	−1.157335	−0.667338
H	−3.287715	0.403720	1.481558
H	−0.702303	1.035028	1.809321
H	−1.428835	1.739890	−1.061306
H	−1.124713	−1.383431	−1.802618
Cl	1.344923	−2.102934	0.088430

TS38

0 2

C	1.398785	2.131345	−0.002635
C	1.911878	0.808696	0.203797
C	1.099528	−0.257743	−0.002714
C	−0.836393	1.242435	−0.488865
C	0.124047	2.350220	−0.359171
H	2.079182	2.962825	0.102441
H	2.938054	0.660890	0.495121
H	−0.221457	3.350754	−0.571368
C	−2.378605	−1.167694	−0.255024
C	−2.473068	−0.196066	0.784222
C	−1.470154	0.761725	0.802036
C	−0.265459	−0.116074	−0.361752
C	−1.143870	−1.203005	−0.805869
H	−3.165700	−1.862854	−0.495911
H	−3.150754	−0.305485	1.616651

H	−1.172978	1.272718	1.705137
H	−1.562568	1.353890	−1.291163
H	−0.805111	−1.928335	−1.528213
Cl	1.721247	−1.860437	0.169597

TS39

0 2

C	−2.012600	−1.746502	−0.036005
C	−0.659497	−1.566228	0.002482
C	−0.105128	−0.274312	0.016917
C	−2.392632	0.621780	−0.039389
C	−2.885760	−0.640557	−0.094573
H	−2.416860	−2.746428	−0.057623
H	0.001254	−2.417361	0.001792
H	−3.947617	−0.804299	−0.193112
C	0.884537	2.363483	−0.092520
C	1.769052	1.267990	−0.035900
C	1.279516	−0.005037	0.002189
C	−0.990078	0.853534	0.131454
C	−0.456145	2.169914	−0.038881
H	1.289105	3.358880	−0.188089
H	−1.142845	3.001552	−0.075638
H	−1.015281	0.879289	1.789177
H	2.834499	1.427991	−0.057737
H	−3.049253	1.477681	−0.076973
Cl	2.403494	−1.320100	−0.002143

TS40

0 2

C	2.565190	0.412553	0.326031
C	2.488522	−0.908444	−0.212883
C	1.310755	−1.449866	−0.565354
C	0.191710	0.724234	0.021010
C	1.460812	1.222692	0.343484
H	3.524198	0.815860	0.609319
H	3.405029	−1.448277	−0.396385
H	1.553050	2.273528	0.569037
C	−2.242834	−1.132604	−0.113138
C	−1.785541	−0.827117	1.200132
C	−0.419043	−0.642935	1.169480
C	0.033765	−0.769264	−0.256262
C	−1.202735	−1.129114	−0.979703
H	−3.273461	−1.301425	−0.378466
H	−2.407155	−0.729552	2.073489

H	0.261972	−0.576957	1.998148
H	−1.242102	−1.280455	−2.044723
Cl	−1.028307	1.862012	−0.409938
H	1.256087	−2.407741	−1.058258

TS41

0 2

C	2.546886	0.346483	0.337202
C	2.480887	−0.977440	−0.205367
C	1.282979	−1.501774	−0.549926
C	0.100458	0.585876	0.183017
C	1.445469	1.092385	0.504939
H	3.389250	−1.538690	−0.354397
C	−2.268166	−1.129351	−0.053692
C	−1.870960	−0.642498	1.210595
C	−0.568778	−0.133049	1.303222
C	0.068344	−0.782913	−0.355149
C	−1.207758	−1.278465	−0.881980
H	−0.054798	−0.103149	2.251888
H	1.214485	−2.486334	−0.988092
Cl	−0.860172	1.862667	−0.662619
H	3.509871	0.761001	0.593973
H	−2.468958	−0.762725	2.100258
H	1.505781	2.104029	0.875687
H	−3.278224	−1.409175	−0.300646
H	−1.250649	−1.705829	−1.871321

TS42

0 2

C	−0.714768	2.074790	0.499509
C	0.066249	1.656854	−0.570748
C	−0.377210	0.306828	−0.971922
C	−1.533246	0.059286	−0.093218
C	−1.694731	1.087345	0.777412
H	−0.580270	2.989019	1.050693
H	−2.422959	1.124731	1.569235
C	2.490733	−0.777045	1.123185
C	3.113139	−0.592436	−0.170989
C	2.180017	−0.123957	−1.042531
C	0.943250	−0.012849	−0.328379
C	1.188328	−0.424065	1.035925
H	2.992236	−1.137425	2.004997
H	4.146098	−0.797283	−0.396718
H	0.450721	−0.440652	1.818037

H	−0.450075	0.029501	−2.014715
H	2.318746	0.104800	−2.085362
Cl	−2.423849	−1.391002	−0.140046
H	0.780369	2.245848	−1.114846

TS43
0 2

C	−0.575569	2.091908	0.293891
C	0.509827	1.615662	−0.397089
C	−0.336012	−0.122147	−0.905306
C	−1.516308	0.031825	−0.074250
C	−1.601424	1.158833	0.658690
H	−0.727356	3.154468	0.409409
H	1.138377	2.268847	−0.982036
H	−2.401984	1.374962	1.344711
C	3.060313	−0.764163	−0.227937
C	2.481308	−0.867830	1.109634
C	1.254223	−0.340557	1.102687
C	0.957672	0.161917	−0.270449
C	2.183413	−0.168969	−1.040961
H	4.040180	−1.118789	−0.501938
H	2.981173	−1.301740	1.959992
H	2.306574	0.054170	−2.087808
H	0.564221	−0.256880	1.924076
Cl	−2.706069	−1.206787	−0.095598
H	−0.402675	−0.438533	−1.934683

TS44
0 2

C	1.466908	1.356863	−0.065609
C	1.562350	−0.064199	0.002477
C	0.502256	−0.878878	−0.132834
C	−0.900586	1.178462	−0.507483
C	0.263347	1.933945	−0.396819
H	2.362053	1.947108	0.033666
H	0.222874	2.989658	−0.618590
C	−3.115070	−0.812104	−0.203192
C	−2.857336	−0.035015	0.960514
C	−1.507403	0.259532	1.010613
C	−0.858936	−0.309429	−0.207479
C	−1.963501	−1.016427	−0.890011
H	−4.090285	−1.155967	−0.506362
H	−3.595167	0.296912	1.670749
H	−0.951065	0.691670	1.822509

H	−1.846441	−1.534598	−1.826461
H	0.618258	−1.949732	−0.154259
H	−1.794907	1.571049	−0.961186
Cl	3.148383	−0.737212	0.183463

TS45
0 2

C	1.432709	1.339781	−0.210719
C	1.579304	−0.077907	−0.034518
C	0.523049	−0.916028	−0.159865
C	−0.995123	1.058594	−0.499695
C	0.231103	1.873919	−0.468801
H	2.317496	1.955566	−0.176497
H	0.145942	2.931164	−0.671355
C	−3.069670	−0.889325	−0.072434
C	−2.820492	0.078162	0.942769
C	−1.607052	0.746160	0.851183
C	−0.776014	−0.398233	−0.397166
C	−1.937772	−1.230899	−0.732155
H	−4.023759	−1.367349	−0.221431
H	−3.425739	0.148752	1.833317
H	−1.110751	1.162556	1.714598
H	0.655302	−1.983690	−0.078948
H	−1.735251	1.368525	−1.233849
H	−1.862349	−2.017779	−1.466475
Cl	3.157580	−0.688417	0.293472

TS46
0 2

C	−2.955285	−1.170195	−0.043452
C	−1.646400	−1.555883	0.005474
C	−0.613786	−0.601099	0.028809
C	−2.333375	1.151201	−0.050584
C	−3.298221	0.198078	−0.107684
H	−3.735498	−1.914723	−0.071093
H	−1.386210	−2.603963	0.005187
H	−4.333440	0.482686	−0.214478
C	1.382104	1.369898	−0.076394
C	1.707834	−0.001211	−0.007061
C	0.743803	−0.964552	0.023733
C	−0.962536	0.785977	0.135080
C	0.081464	1.750053	−0.036275
H	2.173469	2.095314	−0.172639
H	−0.183273	2.795173	−0.086433

H	−0.981707	0.830547	1.793830
H	1.017555	−2.008073	0.022229
H	−2.583633	2.200368	−0.095282
Cl	3.375243	−0.450055	−0.024188

TS47

0 2

C	−1.690258	1.229571	0.274802
C	−0.671209	2.223736	0.185089
C	0.610634	1.913585	−0.084116
C	−0.089045	−0.491030	−0.314723
C	−1.387241	−0.071496	−0.046686
H	−2.706630	1.508924	0.495591
H	−0.957714	3.258060	0.300492
C	2.874451	−0.900023	−0.363522
C	2.268714	−1.167890	0.896344
C	1.187938	−0.323314	1.060007
C	1.046103	0.504613	−0.176697
C	2.202813	0.089344	−1.001562
H	3.722059	−1.434007	−0.760541
H	2.578621	−1.926287	1.594663
H	0.600748	−0.157952	1.945112
H	2.401100	0.482641	−1.983962
H	1.358344	2.680438	−0.209659
H	0.113766	−1.442374	−0.775628
Cl	−2.660453	−1.236001	−0.187040

TS48

0 2

C	−1.667670	1.256316	0.255665
C	−0.647123	2.260519	0.325556
C	0.642017	1.952117	0.035333
C	0.017969	−0.454367	−0.333984
C	−1.359168	−0.002460	−0.086185
H	−2.695038	1.523187	0.446120
H	−0.925270	3.268106	0.588873
C	2.859300	−0.871022	−0.288599
C	2.082374	−1.338230	0.808993
C	0.802746	−0.806342	0.909780
C	1.023616	0.628458	−0.299553
C	2.342097	0.258043	−0.827481
H	3.799358	−1.307287	−0.583145
H	2.508337	−1.910857	1.618212
H	0.277210	−0.735397	1.849669

H	1.406452	2.714433	0.047175
H	0.119192	−1.180513	−1.137129
H	2.812366	0.845960	−1.600298
Cl	−2.581151	−1.206814	−0.248625

TS49
0 2

C	2.900212	−0.599237	−0.039949
C	2.386140	0.663671	−0.003336
C	0.996457	0.884953	0.018305
C	0.691700	−1.553817	−0.031860
C	2.037877	−1.714682	−0.091170
H	3.968154	−0.748897	−0.068250
H	3.043094	1.520775	−0.014825
H	2.453555	−2.705533	−0.189040
C	−1.774523	1.264759	−0.072024
C	−0.899725	2.367652	−0.043362
C	0.451124	2.179396	−0.010665
C	0.123932	−0.250895	0.142314
C	−1.280498	0.002093	−0.013263
H	−2.838835	1.417826	−0.146935
H	0.096530	−0.304144	1.805812
H	1.123073	3.024345	−0.028492
H	−1.310816	3.364147	−0.077083
H	0.031024	−2.403668	−0.064929
Cl	−2.374233	−1.331072	−0.020365

TS50
0 2

C	2.801756	0.160520	0.097438
C	1.891838	−0.097710	1.041877
C	0.842158	−1.001176	0.480078
C	1.257530	−1.151705	−0.948348
C	2.400418	−0.485657	−1.145466
H	3.691039	0.756734	0.219016
H	1.897826	0.246862	2.062468
H	0.699058	−1.723489	−1.670142
H	2.948951	−0.427569	−2.071300
C	−2.624755	−1.063349	−0.346861
C	−2.320229	0.336202	−0.598306
C	−1.134850	0.609910	−0.038373
C	−0.608368	−0.590205	0.657758
C	−1.633048	−1.616844	0.365077
H	−3.519045	−1.559355	−0.685899

H	−2.941817	1.030964	−1.135843
H	−0.730987	−0.337431	1.835360
H	−1.564877	−2.635988	0.706177
Cl	−0.348079	2.124824	−0.031399
H	−0.748442	−0.098081	2.911289
H	0.950932	−1.974571	0.973414

TS51

0 2

C	−3.208057	0.021989	−0.191975
C	−2.154398	0.588033	−0.790910
C	−0.956092	−0.281691	−0.602461
C	−1.482990	−1.410878	0.224161
C	−2.789325	−1.222775	0.441887
H	−4.213222	0.410596	−0.174233
H	−2.125951	1.516736	−1.335963
H	−3.441377	−1.878867	0.995110
C	1.988395	−0.176106	1.412708
C	1.054678	0.637029	2.184442
C	0.013373	0.978870	1.416155
C	0.216503	0.433940	0.047616
C	1.492245	−0.312613	0.177813
H	2.911413	−0.593555	1.776609
H	1.196654	0.898950	3.219798
H	0.446008	1.332676	−0.650346
O	0.565464	2.619842	−1.339060
H	0.910210	3.125412	−0.591548
H	−0.620586	−0.660346	−1.573345
H	−0.853548	1.552013	1.696869
H	−0.874205	−2.230634	0.566914
Cl	2.182053	−1.174500	−1.125529

TS52

0 2

C	2.289648	−0.327762	1.160621
C	1.044328	−0.847216	0.834488
C	0.903327	−0.812555	−0.634752
C	2.209062	−0.319300	−1.111918
C	2.988833	−0.026032	−0.040644
H	2.653855	−0.161897	2.159715
H	0.339601	−1.314331	1.498778
H	2.447763	−0.155881	−2.148555
H	3.975522	0.404829	−0.084522
C	−0.993602	2.311153	0.070062

C	−2.033612	1.309920	0.150816
C	−1.444908	0.100544	−0.027654
C	−0.040824	0.284927	−0.239607
C	0.196121	1.707313	−0.156625
H	−1.157187	3.369444	0.178882
H	−3.080240	1.489109	0.322183
H	1.159476	2.172934	−0.257913
Cl	−2.203857	−1.438721	−0.032267
H	0.416531	−1.631901	−1.148750

TS53

0 2

C	1.912659	−0.492356	1.138070
C	0.686670	−1.106173	1.018046
C	0.710496	−1.171934	−0.955159
C	1.904191	−0.373913	−1.167736
C	2.507387	0.058135	−0.044318
H	2.472808	−0.589245	2.055846
H	0.355312	−1.828113	1.748682
H	2.261172	−0.173795	−2.166200
H	3.379182	0.690324	−0.026731
C	−2.652847	−0.816376	−0.018382
C	−2.268212	0.587707	−0.013103
C	−0.934074	0.664553	−0.023820
C	−0.342494	−0.711868	−0.033320
C	−1.554636	−1.574565	−0.020880
H	−3.670823	−1.167977	−0.020383
H	−2.944281	1.424965	0.010370
H	−1.497806	−2.650438	−0.030816
Cl	−0.042806	2.118878	0.036976
H	0.557311	−2.105915	−1.475732

TS54

0 2

C	−2.853860	−0.409045	0.227515
C	−2.160664	−1.104425	−0.807585
C	−0.840372	−0.939301	−1.012998
C	−0.796800	0.609613	0.970031
C	−2.153435	0.352243	1.137215
H	−3.909335	−0.580688	0.364958
H	−2.703294	−1.825692	−1.399956
H	−2.645293	0.724326	2.023365
C	1.951901	1.205879	−0.110420
C	0.962328	2.168791	−0.447594

C	−0.259845	1.539828	−0.584191
C	−0.088886	0.090527	−0.268794
C	1.367392	−0.013835	−0.037325
H	2.990065	1.410982	0.086702
H	1.135965	3.226231	−0.548790
H	−1.175654	1.941759	−0.977089
H	−0.302048	−1.531413	−1.736080
Cl	2.119576	−1.493985	0.355167
H	−0.189745	1.030594	1.753926

TS55

0 2

C	−2.840522	−0.598336	0.179842
C	−2.039042	−1.413803	−0.689755
C	−0.701511	−1.205091	−0.775870
C	−0.884657	0.767812	0.777573
C	−2.305437	0.397956	0.902427
H	−3.889633	−0.830617	0.285377
H	−2.502351	−2.216199	−1.241016
H	−2.911501	0.948352	1.606691
C	1.796183	1.328798	−0.125042
C	0.736576	2.168347	−0.561329
C	−0.547780	1.671082	−0.392559
C	−0.086406	−0.143619	−0.066458
C	1.362851	0.055317	0.027624
H	2.825175	1.631608	−0.036209
H	0.915998	3.050754	−1.155779
H	−1.370463	1.978784	−1.019866
H	−0.075021	−1.844752	−1.379145
Cl	2.380837	−1.292821	0.328236
H	−0.407967	1.089244	1.701212

TS56

0 2

C	1.507275	−0.252924	1.435818
C	0.161021	−0.498596	1.178723
C	0.807309	−1.051232	−1.192617
C	2.081399	−0.762438	−0.868727
C	2.446030	−0.305931	0.433380
H	1.811782	−0.059698	2.453298
H	−0.547865	−0.681190	1.968896
H	2.862003	−0.925799	−1.596463
H	3.480665	−0.092190	0.648044
C	−2.588241	−0.760992	0.080471

C	−2.145901	0.578158	0.215953
C	−0.777325	0.607002	−0.010281
C	−0.295216	−0.785991	−0.247716
C	−1.545191	−1.575533	−0.228332
H	−3.604865	−1.083302	0.233713
H	−2.747428	1.434587	0.463676
H	−1.564664	−2.646477	−0.336832
Cl	0.108851	2.021705	−0.379909
H	0.552940	−1.464056	−2.155910

TS57
0 2

C	1.505000	0.007934	1.321938
C	0.065802	−0.211739	1.107485
C	0.727027	−1.428450	−0.977488
C	2.013274	−1.019410	−0.809670
C	2.405190	−0.339429	0.389768
H	1.818128	0.450760	2.254624
H	−0.506342	−0.398388	2.012677
H	2.760916	−1.268626	−1.545492
H	3.453906	−0.145769	0.557069
C	−2.594382	−0.645909	0.100824
C	−2.052926	0.668344	0.053093
C	−0.673005	0.745065	0.194203
C	−0.276616	−1.089119	−0.042933
C	−1.650279	−1.604919	−0.051772
H	−3.652157	−0.841497	0.162912
H	−2.623790	1.532452	−0.245766
H	−1.850777	−2.661424	−0.138104
Cl	0.196481	2.050375	−0.525498
H	0.445418	−2.018094	−1.837149

TS58
0 2

C	−2.102430	−1.562194	0.364283
C	−1.525260	−0.557262	1.021293
C	−0.653544	0.209677	0.074423
C	−0.869098	−0.492324	−1.233294
C	−1.688262	−1.525188	−1.041651
H	−2.775271	−2.291206	0.784672
H	−1.626874	−0.293877	2.060088
H	−0.381953	−0.183054	−2.141337
H	−2.005520	−2.230516	−1.791514
Cl	−1.233574	1.913371	−0.026905

C	2.875402	0.220461	−0.505566
C	2.670286	−1.074964	0.128571
C	1.449710	−1.106373	0.681682
C	0.808248	0.212316	0.478397
C	1.778324	0.973712	−0.338306
H	3.773723	0.515427	−1.022498
H	3.393642	−1.873453	0.142314
H	0.820866	0.746629	1.563890
H	1.610606	1.979810	−0.680733
H	0.711479	1.235511	2.548010
H	0.989809	−1.919754	1.215510

TS59

0 2

C	−2.200833	−1.313852	0.920504
C	−1.545911	−0.161739	1.058999
C	−0.678590	0.050773	−0.143813
C	−0.980787	−1.139011	−1.003907
C	−1.845696	−1.924769	−0.364139
H	−2.887662	−1.741523	1.631945
H	−1.571506	0.534575	1.879285
H	−0.516622	−1.293970	−1.962009
H	−2.230696	−2.865382	−0.721352
Cl	−1.195101	1.565019	−0.989787
C	2.772834	−0.457706	−0.799567
C	2.539398	−1.328878	0.348191
C	1.376896	−0.998463	0.924134
C	0.798693	0.158424	0.193590
C	1.751876	0.399939	−0.919145
H	3.637534	−0.507335	−1.440734
H	3.205534	−2.112687	0.669082
H	0.902809	1.063775	0.927251
H	1.625619	1.170259	−1.659587
O	0.643451	2.267134	1.679292
H	0.161555	2.750199	0.993680
H	0.915255	−1.448624	1.785406

TS60

0 2

C	−1.233480	−1.673965	1.061824
C	−0.709671	−0.393540	1.195489
C	−0.819913	0.275877	−0.113519
C	−1.542400	−0.691107	−0.967425
C	−1.744746	−1.826898	−0.257682

H	−0.407385	0.103705	2.097815
H	−1.785455	−0.499533	−1.997417
C	2.715409	−0.758236	−0.478798
C	2.844746	0.508436	0.214623
C	1.604789	0.993530	0.482420
C	0.656264	0.057003	−0.046944
C	1.404518	−1.036461	−0.639392
H	3.538102	−1.370753	−0.806109
H	3.777670	0.981530	0.470734
H	0.967305	−1.901982	−1.102868
H	1.352407	1.922694	0.961724
H	−1.226815	−2.431225	1.826240
H	−2.196894	−2.727261	−0.639330
Cl	−1.357178	1.952647	−0.206727

TS61

0 2

C	2.012280	−1.721354	−0.274768
C	1.460479	−0.754740	−1.012493
C	0.560252	0.049245	−0.146401
C	0.680918	−0.583194	1.192625
C	1.522898	−1.615198	1.103811
H	2.712095	−2.463857	−0.620791
H	1.619088	−0.543349	−2.055737
H	0.138763	−0.235311	2.054145
H	1.805662	−2.276331	1.905872
Cl	1.382306	1.798852	−0.023668
C	−2.827542	0.403125	0.522027
C	−2.750202	−0.912280	−0.101967
C	−1.602737	−1.011005	−0.780958
C	−0.847057	0.276974	−0.660416
C	−1.724961	1.098960	0.225541
H	−3.651766	0.747879	1.124863
H	−3.505780	−1.676446	−0.020053
H	−0.774091	0.754533	−1.642525
H	−1.481101	2.099558	0.537919
H	1.974383	3.488839	0.054450
H	−1.242428	−1.859200	−1.337785

TS62

0 2

C	−1.050012	2.587600	−0.186217
C	−0.685201	1.575513	−1.013927
C	−0.066615	0.546831	−0.211409

C	−0.070060	1.051952	1.175561
C	−0.661670	2.254873	1.178960
H	−1.547906	3.496114	−0.481333
H	−0.826867	1.514470	−2.078427
H	0.342193	0.505906	2.005666
H	−0.836547	2.881143	2.036871
Cl	−1.794201	−0.774783	−0.258753
C	2.595517	−1.467138	0.561660
C	3.219845	−0.254781	0.048405
C	2.332540	0.420707	−0.690489
C	1.035033	−0.332984	−0.712487
C	1.326139	−1.522480	0.143551
H	3.092343	−2.197473	1.179028
H	0.597925	−2.286828	0.354690
H	4.239547	0.038835	0.237257
O	−2.820192	−2.319483	0.158829
H	−3.128904	−2.091400	1.040590
H	0.798662	−0.653396	−1.730742
H	2.479419	1.359254	−1.197073

TS63
0 2

C	−2.393586	0.699752	−0.007599
C	−1.822121	0.142899	−1.106659
C	−0.837223	−0.866268	−0.676580
C	−0.962528	−0.891208	0.793430
C	−1.874603	0.091351	1.165750
H	−3.105499	1.508769	−0.015932
H	−1.982333	0.428509	−2.131921
H	−0.524180	−1.635518	1.431679
H	−2.121038	0.361749	2.177882
C	2.708429	−0.175583	0.110166
C	2.178058	1.169787	0.103967
C	0.843227	1.111556	−0.106879
C	0.486676	−0.284672	−0.251015
C	1.685803	−1.050310	−0.093680
H	3.744591	−0.433112	0.252422
H	2.758877	2.064644	0.249062
H	1.749440	−2.123367	−0.156113
H	−0.744098	−1.788427	−1.234600
H	0.151444	1.932931	−0.157883

TS64
0 2

C	2.407614	0.579571	−0.127887
C	1.851523	0.398497	1.087969
C	0.698494	−0.484311	1.110481
C	0.662868	−0.911042	−0.829113
C	1.857000	−0.301510	−1.120487
H	3.247864	1.227118	−0.316830
H	2.219116	0.852438	1.995737
H	0.346684	−1.811489	−1.332268
H	2.437383	−0.616230	−1.974766
C	−2.143957	1.130443	−0.274304
C	−2.650562	−0.204429	0.034125
C	−1.618595	−1.023027	0.259166
C	−0.353642	−0.261415	0.108687
C	−0.808736	1.117723	−0.228832
H	−2.763843	1.981134	−0.505873
H	−0.135079	1.936032	−0.412022
H	−1.655599	−2.069996	0.511494
H	0.585100	−1.269963	1.842747
H	−3.693667	−0.472035	0.072956

TS65

0 2

C	−2.464910	−0.397716	0.049102
C	−2.096605	0.947290	0.351534
C	−0.829092	1.382932	0.219495
C	−0.207260	−0.894013	−0.643559
C	−1.546137	−1.253600	−0.517027
H	−3.493763	−0.702016	0.156374
H	−2.870614	1.643365	0.638551
H	−1.866758	−2.205115	−0.914395
C	2.560283	0.195112	−0.261909
C	2.117738	−0.667016	0.779734
C	0.750729	−0.521851	0.926458
C	0.267156	0.445731	−0.102057
C	1.508353	0.880441	−0.776077
H	3.578137	0.264127	−0.609456
H	2.737840	−1.345828	1.339897
H	0.122283	−0.902187	1.711117
H	−0.574442	2.419773	0.373730
H	0.480981	−1.454497	−1.254213
H	1.524801	1.578511	−1.595773

TS66

0 2

C	−2.537936	0.262058	−0.177463
C	−1.528748	1.066759	−0.585375
C	−0.190669	0.580773	−0.229870
C	−0.704672	−1.007933	0.656727
C	−2.072505	−0.778349	0.678198
H	−3.579743	0.446112	−0.382444
H	−1.654901	1.979201	−1.147574
H	−2.696098	−1.219768	1.440383
C	2.439768	−0.389904	−0.001299
C	2.107284	0.935054	0.441391
C	0.840868	1.400987	0.294795
C	0.097238	−0.816048	−0.616684
C	1.515959	−1.203682	−0.536668
H	3.468368	−0.713435	0.056377
H	1.801291	−2.167019	−0.933149
H	−0.431464	−1.174122	−1.497511
H	0.591834	2.414751	0.572558
H	−0.177508	−1.431569	1.498102
H	2.878695	1.567557	0.850742

TS67
0 2

C	2.411633	0.715161	−0.036702
C	1.234199	1.405592	0.000253
C	0.000000	0.731525	0.018895
C	1.244566	−1.386556	−0.039314
C	2.411786	−0.696194	−0.093023
H	3.349422	1.248101	−0.061278
H	1.234939	2.485825	−0.006173
H	3.348028	−1.223775	−0.190975
C	−2.411788	−0.696196	−0.092970
C	−2.411640	0.715162	−0.036756
C	−1.234204	1.405590	0.000188
C	0.000003	−0.700154	0.132929
C	−1.244570	−1.386553	−0.039240
H	−3.348031	−1.223792	−0.190832
H	−1.234500	−2.465490	−0.077889
H	0.000129	−0.733574	1.787308
H	−1.234936	2.485823	−0.006281
H	−3.349426	1.248102	−0.061384
H	1.234468	−2.465486	−0.078049

TS68
0 2

C	−1.754026	1.284730	0.526086
C	−1.799441	−0.016921	−0.125983
C	−0.680423	−0.242046	−0.816389
C	0.203275	0.953115	−0.666225
C	−0.583450	1.854513	0.229721
C	2.490099	−0.172759	−1.023430
C	3.093939	−1.117709	−0.287954
C	1.587832	0.605725	−0.146853
C	2.597129	−1.047522	1.080965
C	1.695269	−0.059662	1.169998
H	2.911179	−1.696920	1.881311
H	−0.234157	2.819984	0.556302
H	2.646073	0.038453	−2.068084
H	1.136629	0.241328	2.039710
H	0.320051	1.441231	−1.640110
H	−2.547735	1.685879	1.132867
H	3.832508	−1.820352	−0.637459
H	2.137609	1.668878	−0.011315
H	2.551113	2.709391	0.066604
H	−0.424707	−1.121920	−1.379380
Cl	−3.142928	−1.071455	0.024702

TS69

0 2

C	−1.730765	1.431580	0.389834
C	−0.488732	1.750525	0.017644
C	0.094074	0.637908	−0.792477
C	−0.990754	−0.389363	−0.806076
C	−2.027604	0.101621	−0.124956
H	−2.419956	2.024869	0.966153
H	0.048924	2.657113	0.239619
C	2.509097	−1.913521	−0.248001
C	2.041745	−1.646268	1.109161
C	1.381408	−0.482581	1.123532
C	1.411539	0.105086	−0.244161
C	2.130158	−0.910074	−1.051037
H	3.069607	−2.785780	−0.542388
H	2.201916	−2.298707	1.951653
H	2.105994	1.027412	−0.204819
H	2.329962	−0.807872	−2.104351
O	2.886780	2.254927	0.141721
H	3.376545	1.867359	0.878749
H	0.272149	0.998285	−1.810682
H	0.895356	−0.013007	1.962024

H	−0.916292	−1.351136	−1.281485
Cl	−3.531731	−0.674555	0.151234

TS70
0 2

C	1.569206	0.034071	−0.144948
C	0.510819	0.208118	−1.022241
C	−0.182249	1.448507	−0.623534
C	0.633735	1.990174	0.480970
C	1.639606	1.128398	0.761313
H	0.314844	−0.363113	−1.909485
H	0.397871	2.894842	1.013820
H	2.360233	1.214026	1.556619
C	−2.465597	−0.936517	1.083689
C	−3.053727	−0.846550	−0.235619
C	−2.274296	−0.042904	−1.008387
C	−1.184191	0.409461	−0.196183
C	−1.338563	−0.189530	1.111545
H	−2.866398	−1.510297	1.901669
H	−3.960846	−1.337856	−0.544890
H	−0.666416	−0.050390	1.938916
H	−0.550520	2.126795	−1.381971
H	−2.441372	0.235349	−2.034993
Cl	2.604950	−1.319336	−0.104666

TS71
0 2

C	1.068941	−0.620515	1.173792
C	−0.083539	−1.298623	0.794304
C	−0.192769	−1.244094	−0.676140
C	1.036551	−0.564032	−1.117689
C	1.727809	−0.199860	−0.009416
H	−0.711409	−1.893786	1.430597
H	1.283192	−0.326346	−2.136670
C	−2.480266	1.582205	0.098840
C	−3.382852	0.450103	0.107434
C	−2.662819	−0.687167	−0.089386
C	−1.291278	−0.306150	−0.244630
C	−1.221929	1.135155	−0.106145
H	−2.774205	2.608272	0.239049
H	−4.449371	0.509014	0.245762
H	−0.319802	1.717929	−0.157661
H	−0.545877	−2.104267	−1.228852
H	−3.039069	−1.694134	−0.150026

H	1.397124	−0.416753	2.177069
Cl	3.179549	0.712819	−0.000298

TS72
0 2

C	−1.042257	−0.697167	−1.104010
C	0.275668	−1.038917	−0.940687
C	0.266790	−0.965962	1.032853
C	−1.054998	−0.391797	1.212019
C	−1.696598	−0.155522	0.053786
H	−1.608619	−0.999865	−1.970490
H	0.754724	−1.753931	−1.590988
H	−1.467634	−0.190013	2.187270
C	3.375844	0.307343	−0.051522
C	2.546159	1.509078	−0.102423
C	1.258758	1.161884	−0.026859
C	1.170378	−0.323465	0.068662
C	2.587349	−0.766389	0.048097
H	4.452717	0.302793	−0.087139
H	2.928683	2.512534	−0.191696
H	2.890120	−1.798787	0.106840
H	0.602419	−1.820426	1.601602
H	0.398150	1.807453	−0.042219
Cl	−3.239595	0.594454	−0.067805

TS73
0 2

C	0.879058	−0.967214	−0.625983
C	−0.497168	−0.775201	−0.688540
C	−0.138657	1.538302	0.223465
C	1.177739	1.273484	0.299468
C	1.685943	−0.010400	−0.056341
H	1.316821	−1.849584	−1.065476
H	−1.123785	−1.396553	−1.306210
H	1.878960	2.044213	0.577698
C	−3.362255	−0.063130	−0.201594
C	−2.780717	−0.894385	0.794065
C	−1.438207	−0.577971	0.911325
C	−1.118124	0.478214	−0.091590
C	−2.423660	0.770610	−0.717660
H	−4.390352	−0.116356	−0.520135
H	−3.289120	−1.668337	1.343058
H	−2.554437	1.486317	−1.511355
H	−0.512855	2.531435	0.415140

Cl	3.383288	−0.280592	0.077566
H	−0.744835	−0.894930	1.668970

TS74

0 2

C	0.862994	−0.929028	−0.588719
C	−0.595334	−0.725068	−0.637449
C	−0.139237	1.558944	0.287571
C	1.180211	1.269499	0.414723
C	1.662735	0.001195	−0.050920
H	1.278430	−1.836653	−0.997293
H	−1.082704	−1.141769	−1.515802
H	1.878783	1.981267	0.820883
C	−3.341939	−0.016404	−0.144438
C	−2.725446	−0.989585	0.694757
C	−1.340562	−1.034739	0.646661
C	−1.060616	0.616471	−0.232485
C	−2.458758	0.919814	−0.562758
H	−4.402124	0.025353	−0.332134
H	−2.716077	1.807219	−1.120103
H	−0.511732	2.528540	0.583203
H	−0.745479	−1.389949	1.474244
H	−3.270274	−1.517068	1.462416
Cl	3.370993	−0.263737	0.038995

TS75

0 2

C	3.299954	0.225365	−0.090024
C	2.321823	1.177207	−0.041976
C	0.964969	0.815109	0.019376
C	1.666246	−1.538348	−0.019776
C	2.961896	−1.145307	−0.114959
H	4.336320	0.519271	−0.147426
H	2.577883	2.226061	−0.073192
H	3.742782	−1.882268	−0.221487
C	−1.705312	0.004605	0.008624
C	−1.383773	1.378102	0.027421
C	−0.074750	1.761535	0.014643
C	0.627069	−0.571194	0.167086
C	−0.746485	−0.951915	0.043321
H	0.684691	−0.575986	1.821251
H	0.175284	2.811565	−0.017178
H	−2.178529	2.105716	0.015785
H	1.400041	−2.584248	−0.033805

H	−1.006495	−1.998217	0.029063
Cl	−3.371871	−0.444285	−0.085613

TS76
0 2

C	−3.041638	−1.095522	0.376032
C	−2.487824	−0.062767	1.020342
C	−1.682365	0.746874	0.057467
C	−1.840492	−0.005623	−1.225440
C	−2.635373	−1.061703	−1.024278
H	−3.688948	−1.840937	0.808501
H	−2.595990	0.185601	2.063286
H	−1.363321	0.285813	−2.145940
H	−2.932754	−1.784181	−1.766477
C	1.802036	1.262085	−0.560734
C	1.777661	−0.046292	0.077703
C	0.594247	−0.268405	0.661373
C	−0.230795	0.939734	0.463036
C	0.610382	1.837872	−0.362307
H	2.650877	1.664722	−1.086215
H	−0.282546	1.482655	1.534158
H	0.298374	2.812708	−0.697569
H	−0.453407	2.009045	2.519499
H	−2.136864	1.738616	−0.044095
Cl	3.119563	−1.110769	0.042622
H	0.276976	−1.148503	1.191125

TS77
0 2

C	2.945340	−1.017788	−0.886525
C	2.483584	0.219426	−0.674510
C	1.651639	0.228927	0.565632
C	1.686130	−1.199397	1.006645
C	2.446437	−1.902556	0.160474
H	3.585708	−1.327505	−1.696283
H	2.659377	1.100189	−1.269413
H	1.162939	−1.567179	1.873273
H	2.662539	−2.956340	0.227307
C	−1.819984	0.384621	1.314889
C	−1.800237	−0.183241	−0.028736
C	−0.610195	0.002924	−0.605773
C	0.238525	0.757144	0.350489
C	−0.618900	0.921208	1.552932
H	−2.673651	0.359030	1.970382

H	0.387472	1.818075	−0.073705
H	−0.302173	1.430440	2.447353
O	0.619381	3.111610	−0.810847
H	−0.224784	3.125323	−1.280779
H	2.134761	0.860655	1.317960
H	−0.289879	−0.328185	−1.577494
Cl	−3.157141	−0.980883	−0.704996

TS78

0 2

C	−2.389142	−0.647346	1.173714
C	−1.928085	0.627072	0.853690
C	−1.862875	0.733581	−0.616763
C	−2.408639	−0.548410	−1.097547
C	−2.684838	−1.340769	−0.028865
H	−2.477182	−1.045686	2.169567
H	−1.787199	1.446842	1.533273
H	−2.487810	−0.816408	−2.136896
H	−3.038752	−2.357300	−0.079741
C	1.702008	1.432405	0.081568
C	1.713407	−0.005863	−0.043240
C	0.462001	−0.476909	−0.235578
C	−0.403436	0.675181	−0.245728
C	0.413406	1.839321	−0.034169
H	2.573931	2.042660	0.237524
H	0.048936	2.851774	0.000886
H	−2.147171	1.651107	−1.114586
H	0.160846	−1.500419	−0.356734
Cl	3.145386	−0.941303	0.053189

TS79

0 2

C	−2.461229	−0.416066	1.174734
C	−1.604570	0.641063	0.996983
C	−1.658996	0.595441	−0.990471
C	−2.401634	−0.644721	−1.129868
C	−2.738067	−1.241868	0.032005
H	−3.034600	−0.507855	2.084784
H	−1.593912	1.483333	1.670946
H	−2.658621	−1.028500	−2.105430
H	−3.261313	−2.181347	0.097897
C	1.639221	1.420117	−0.044114
C	1.670434	−0.037984	−0.011953
C	0.437828	−0.543789	−0.032285

C	−0.512997	0.603242	−0.069493
C	0.362014	1.802824	−0.079657
H	2.514851	2.045976	−0.040828
H	−0.015378	2.811575	−0.108710
H	−1.905031	1.486015	−1.549429
Cl	3.142160	−0.917731	0.052268
H	0.145274	−1.577329	−0.013073

TS80
0 2

C	−3.273363	−0.304883	−0.159643
C	−2.879793	1.007588	0.240402
C	−1.588049	1.386939	0.259165
C	−0.980214	−0.884742	−0.634750
C	−2.340636	−1.182565	−0.663059
H	−4.320513	−0.561400	−0.173785
H	−3.648061	1.727887	0.478306
H	−2.660099	−2.100915	−1.132461
C	1.755182	0.063211	0.083882
C	1.185964	−0.826747	1.033851
C	−0.179559	−0.619329	1.034471
C	−0.507409	0.411241	0.005842
C	0.810737	0.823453	−0.518002
H	1.731126	−1.549384	1.614315
H	−0.902689	−0.992153	1.736727
H	−1.304094	2.402175	0.487163
H	−0.259109	−1.454371	−1.197154
Cl	3.434449	0.102249	−0.270878
H	0.960653	1.544944	−1.301133

TS81
0 2

C	1.144357	−0.993947	0.842274
C	−0.230977	−1.101623	0.710277
C	−0.551564	0.533481	−0.165672
C	0.839058	0.926146	−0.425534
C	1.730287	0.027025	0.041928
H	1.687051	−1.474987	1.639812
H	−0.851169	−1.482175	1.507422
C	−3.257489	−0.234312	−0.128015
C	−2.368060	−1.102690	−0.634466
C	−0.922001	−0.825868	−0.611632
C	−1.547128	1.417799	0.324982
C	−2.851448	1.046030	0.379377

H	−4.309705	−0.475646	−0.144065
H	−3.596125	1.724788	0.763205
H	−1.239436	2.400352	0.650736
H	−0.362408	−1.207209	−1.462156
H	−2.700061	−2.027631	−1.082104
Cl	3.433535	0.151001	−0.171025
H	1.091545	1.823243	−0.966543

TS82
0 2

C	−1.932116	−1.244181	0.907883
C	−0.813067	−0.424825	1.015720
C	−1.812138	1.031091	−0.772989
C	−2.846998	0.170930	−0.841878
C	−2.893576	−1.015787	−0.051995
H	−2.059026	−2.044592	1.621178
H	−0.154706	−0.465085	1.867723
H	−3.692628	0.408694	−1.469796
H	−3.745217	−1.671910	−0.132931
C	1.561016	1.352798	0.507691
C	1.612541	0.083077	−0.133015
C	0.344090	−0.334893	−0.468932
C	−0.609031	0.704288	0.022222
C	0.271356	1.755357	0.579629
H	2.420284	1.870770	0.898155
H	−0.098509	2.658619	1.033962
H	−1.818487	1.962869	−1.316228
H	0.060906	−1.162451	−1.092162
Cl	3.046760	−0.824944	−0.352700

TS83
0 2

C	−1.958892	−1.220739	0.834148
C	−0.753266	−0.382474	0.975970
C	−1.770906	1.059947	−0.814104
C	−2.800758	0.188019	−0.951645
C	−2.889482	−0.963677	−0.096664
H	−2.078128	−2.053592	1.511134
H	−0.413435	−0.240595	1.999595
H	−3.577242	0.371468	−1.676950
H	−3.752158	−1.607032	−0.183523
C	1.552335	1.293425	0.577889
C	1.587120	−0.064070	0.151635
C	0.371421	−0.717211	0.025220

C	−0.730888	0.827598	0.125032
C	0.306987	1.807398	0.455628
H	2.430322	1.846227	0.864801
H	0.056591	2.843812	0.621485
H	−1.719040	1.953175	−1.419079
H	0.228286	−1.530838	−0.667957
Cl	3.020164	−0.738348	−0.514598

Table S5. Cartesian coordinates for the intermediates and products involved in PCN formation from cross−condenstaion of PhR with 2−CPR/3−CPR.

IM1

0 1

C	−1.167854	1.944439	1.112373
C	−2.400339	1.228707	0.880434
C	−2.458324	0.144099	0.092994
C	−1.249245	−0.402429	−0.532975
C	−0.015880	0.469358	−0.484212
C	−0.042102	1.582117	0.497703
H	0.521928	−1.583871	1.281865
C	1.407046	−1.126919	0.862638
C	2.599833	−1.295367	1.433399
C	1.249838	−0.384045	−0.415245
C	3.783963	−0.707866	0.843950
C	2.459101	0.409943	−0.858195
C	3.735820	0.075274	−0.244389
O	2.351699	1.276060	−1.701666
H	4.613882	0.547851	−0.655058
H	4.735973	−0.898579	1.318275
H	2.702687	−1.876480	2.336188
H	0.017256	0.952866	−1.470628
H	−3.300357	1.568655	1.370111
Cl	−3.911644	−0.733504	−0.135498
O	−1.229084	−1.479384	−1.080275
H	0.875643	2.131914	0.647174
H	−1.186106	2.783938	1.788967
H	1.124962	−1.154000	−1.188759

IM2

0 1

C	−1.778605	2.223654	−0.319872
C	−2.847423	1.313593	0.027154
C	−2.679560	−0.015480	0.023199
C	−1.391457	−0.623547	−0.337519
C	−0.242322	0.325336	−0.650645
C	−0.563871	1.773117	−0.627270
H	1.216100	1.759100	1.192834
C	1.573623	0.746186	1.047559
C	2.727405	0.338245	1.838976
C	0.929230	−0.059865	0.207769
C	3.821763	−0.279203	1.380260
C	3.525254	−0.414639	−1.094385

C	4.185316	−0.641011	0.022662
O	3.005041	−0.273014	−2.117515
H	5.142980	−1.108261	−0.153062
H	4.587385	−0.528161	2.099592
H	2.708213	0.591461	2.889879
H	−3.811218	1.720452	0.293048
O	−1.226258	−1.816460	−0.386270
H	0.230369	2.457301	−0.888389
H	−1.995694	3.280234	−0.326013
H	0.037185	0.068698	−1.680331
Cl	−3.948000	−1.090772	0.433577
H	1.214292	−1.100226	0.124597

IM3

0 1

C	−0.580657	2.193991	−0.001545
C	−1.860082	1.544295	0.162321
C	−2.013655	0.221770	−0.000530
C	−0.873326	−0.640925	−0.332749
C	0.402483	0.063931	−0.748946
C	0.488410	1.504240	−0.398173
C	1.960358	−0.571901	1.138287
C	3.216929	−0.132713	1.271598
C	1.640681	−0.727817	−0.312294
C	3.804646	0.041314	−0.051884
C	2.899434	−0.289581	−0.980343
H	3.034145	−0.270839	−2.049589
H	4.813370	0.373050	−0.236679
O	−0.945278	−1.845649	−0.326115
H	1.455473	1.974908	−0.505086
H	1.265994	−0.802209	1.929106
H	−2.713270	2.138383	0.453288
H	−0.511957	3.246998	0.221001
H	3.728823	0.060710	2.199959
H	0.357452	0.014006	−1.845964
Cl	−3.518487	−0.554735	0.264791
H	1.435141	−1.778940	−0.533016

IM4

0 1

C	0.856469	2.005617	0.023493
C	2.047688	1.427533	0.201749
C	2.418357	0.033782	0.050860
C	1.655308	−1.025137	−0.155572

C	−0.841074	0.275762	0.404492
C	−0.379601	1.307637	−0.299153
C	−2.837985	−0.133954	−1.119986
C	−4.116380	0.076938	−0.789485
C	−2.068377	−0.517201	0.098213
C	−4.280793	−0.114671	0.646787
C	−3.103333	−0.453232	1.181272
H	−5.210950	−0.000141	1.178847
O	1.079707	−2.019254	−0.293415
H	−0.958147	1.677493	−1.136800
H	−2.409130	−0.081901	−2.106780
H	2.893069	2.051280	0.451800
H	−2.893530	−0.656185	2.218043
H	−0.301461	−0.026043	1.295582
H	0.816604	3.083595	0.095750
H	−4.912368	0.339017	−1.467268
Cl	4.117220	−0.350909	0.021917
H	−1.756156	−1.566084	−0.010471

IM5

0 1

C	2.547022	−1.080533	0.340835
C	1.644648	−1.575712	−0.511717
C	0.625906	−0.531188	−0.840233
C	1.110247	0.625563	−0.023015
C	2.219503	0.304087	0.648079
H	3.395459	−1.606427	0.746737
H	1.614975	−2.573586	−0.916917
H	2.777317	0.955046	1.298763
C	−2.765730	0.114192	−0.020494
C	−2.267194	−0.542244	1.182086
C	−1.124671	−1.176278	0.897149
C	−0.808917	−0.993348	−0.550628
C	−1.914651	−0.109050	−1.026893
H	−3.674149	0.691596	−0.072508
H	−2.753160	−0.510165	2.143499
H	−0.928339	−1.960814	−1.053253
H	−1.999421	0.249593	−2.039318
H	0.696658	−0.271281	−1.900588
H	−0.505777	−1.743225	1.572289
Cl	0.339968	2.150372	−0.020572

IM6

0 1

C	3.507171	0.942848	1.273315
C	4.392891	0.399959	0.262969
C	3.974907	−0.465581	−0.670335
C	2.586673	−0.906211	−0.717661
C	1.624914	−0.338762	0.319508
C	2.217248	0.611735	1.295978
H	0.431438	2.157528	0.283907
C	−0.096667	1.403189	−0.287399
C	−1.326127	1.810798	−0.952614
C	0.405026	0.174150	−0.383856
C	−2.499336	1.179113	−0.854900
C	−2.078950	−0.759368	0.678341
C	−2.853591	0.032161	−0.042556
O	−1.489402	−1.528607	1.309769
H	−3.342596	1.567804	−1.406301
H	−1.296739	2.711330	−1.549803
H	5.427363	0.711786	0.273852
O	2.186230	−1.696657	−1.542777
H	1.558443	1.011977	2.053594
H	3.915440	1.618237	2.008921
H	4.633849	−0.865779	−1.424546
H	−0.059297	−0.547804	−1.043710
Cl	−4.544718	−0.343976	0.132792
H	1.292723	−1.219573	0.885938

IM7

0 1

C	−2.026846	0.200246	−1.832031
C	−3.186051	−0.017273	−0.993253
C	−3.087152	−0.290054	0.316823
C	−1.783881	−0.352191	0.964752
C	−0.570458	−0.407712	0.059743
C	−0.798698	0.051001	−1.335772
C	0.625383	1.737099	0.679902
C	1.727564	2.185866	0.068347
C	0.645773	0.244322	0.719221
C	2.552145	1.058919	−0.348309
C	1.928123	−0.061725	0.024097
H	3.499414	1.122766	−0.855453
O	−1.659520	−0.396191	2.169958
H	0.077722	0.222670	−1.945477
H	−0.169755	2.326234	1.104106
H	−4.164399	0.075601	−1.442814
H	−2.176442	0.497256	−2.858108

H	1.989222	3.217972	−0.096267
H	−3.952731	−0.397325	0.951094
H	0.674146	−0.105253	1.754101
Cl	2.452768	−1.670411	−0.229803
H	−0.353481	−1.484392	−0.005326

IM8

0 1

C	2.242921	−1.036844	−1.474987
C	3.296386	−0.285878	−1.134971
C	3.528527	0.502555	0.061392
C	2.752987	0.656807	1.114949
C	0.300397	−0.170331	−0.234314
C	1.010242	−1.188992	−0.713356
C	−1.444013	−1.658083	0.888845
C	−2.718630	−1.773304	0.502588
C	−0.947671	−0.284661	0.580471
C	−3.167061	−0.528608	−0.106811
C	−2.149705	0.333888	−0.070315
H	−4.147726	−0.339503	−0.507835
O	2.132576	0.870006	2.067570
H	0.645274	−2.198323	−0.566889
H	−0.847868	−2.406526	1.382584
H	4.134404	−0.271037	−1.815568
H	2.325491	−1.616943	−2.383720
H	−3.342740	−2.643097	0.625288
H	4.479018	1.002295	0.177498
H	−0.780143	0.244174	1.528179
Cl	−2.139584	1.942771	−0.647820
H	0.620332	0.842508	−0.448090

IM9

0 1

C	−1.977757	−0.038006	1.935070
C	−2.950652	−0.806954	1.186453
C	−2.781620	−1.110530	−0.106472
C	−1.560084	−0.730018	−0.797691
C	−0.627573	0.269854	−0.101245
C	−0.895063	0.469830	1.349103
H	0.371872	−2.252278	−0.205744
C	1.155783	−1.516868	−0.115649
C	2.409044	−1.897921	0.131481
C	0.824853	−0.088112	−0.408102
C	3.475747	−0.922916	0.202501

C	1.851273	0.880835	0.144505
C	3.229519	0.393531	0.168886
O	1.565266	1.989366	0.532654
H	4.003521	1.132680	0.301381
H	4.489296	−1.277104	0.322349
H	2.645049	−2.941239	0.269436
H	−3.838957	−1.142276	1.701526
O	−1.259853	−1.164600	−1.884770
H	−0.190301	1.086954	1.883237
H	−2.159151	0.140714	2.983236
H	0.927868	0.001089	−1.499743
Cl	−1.065934	1.819604	−0.929715
H	−3.492445	−1.696282	−0.666632

IM10
0 1

C	2.774186	−0.841518	−1.404831
C	3.576317	−0.938602	−0.201279
C	3.131254	−0.517578	0.987734
C	1.793034	0.040089	1.135871
C	0.934099	0.199198	−0.130589
C	1.550866	−0.318990	−1.379629
H	−0.414484	−1.819537	−1.305933
C	−0.971702	−1.370901	−0.492721
C	−2.217266	−2.043144	−0.153829
C	−0.418962	−0.351964	0.165870
C	−3.372531	−1.531285	0.290596
C	−3.268228	0.913915	−0.092639
C	−3.795856	−0.156857	0.465565
O	−2.844233	1.878201	−0.560184
H	−4.698908	0.051489	1.018789
H	−4.148499	−2.240795	0.538356
H	−2.198382	−3.115555	−0.290864
H	4.567336	−1.360264	−0.283206
O	1.340302	0.360999	2.207006
H	0.963728	−0.216179	−2.279754
H	3.196717	−1.187458	−2.335201
H	−0.884438	0.077033	1.042176
H	3.722827	−0.582055	1.886788
Cl	0.802016	1.999150	−0.370967

IM11
0 1

C	1.387734	−1.238916	−1.572373

C	2.503381	−1.327756	−0.652172
C	2.481287	−0.748515	0.554517
C	1.297628	−0.029324	1.005482
C	0.239246	0.317051	−0.046563
C	0.325306	−0.486170	−1.295206
C	−1.579059	−1.107799	0.891180
C	−2.751156	−1.354808	0.295477
C	−1.152230	0.290477	0.572409
C	−3.166739	−0.175932	−0.453432
C	−2.242378	0.782334	−0.318177
H	−2.255998	1.767615	−0.751540
H	−4.082546	−0.098618	−1.016365
O	1.133287	0.303162	2.154669
H	−0.513158	−0.407815	−1.971452
H	−1.018820	−1.772611	1.526868
H	3.368363	−1.899916	−0.954337
H	1.432149	−1.798232	−2.493611
H	−3.314518	−2.271278	0.360145
H	−1.099951	0.881500	1.490508
H	3.291629	−0.838812	1.259693
Cl	0.651084	2.028766	−0.504359

IM12

0 1

C	−1.603457	0.161314	1.847962
C	−2.824615	0.113823	1.304955
C	−3.239835	−0.345946	−0.003939
C	−2.490339	−0.920738	−0.922777
C	0.221938	0.296189	0.135268
C	−0.345687	−0.224757	1.220373
C	2.144495	−1.365482	0.112506
C	3.413045	−1.097471	0.439980
C	1.519210	−0.140785	−0.468828
C	3.701650	0.302756	0.153213
C	2.612382	0.880569	−0.360422
H	4.649624	0.782518	0.333091
O	−1.883357	−1.433921	−1.760775
H	0.235809	−0.990050	1.719674
H	1.641067	−2.314712	0.186250
H	−3.650121	0.433531	1.923181
H	2.494202	1.905302	−0.667380
H	−1.538589	0.455704	2.886604
H	4.124572	−1.800379	0.841764
H	1.335181	−0.327839	−1.534388

H	−4.275686	−0.257802	−0.293509
Cl	−0.505996	1.625192	−0.709343

IM13
0 1

C	2.036069	−1.611351	−0.281617
C	1.525260	−1.533017	1.091049
C	0.726114	−0.473206	1.203230
C	0.630212	0.212041	−0.125266
C	1.538815	−0.601592	−0.994429
C	−1.753239	1.054898	0.207313
C	−2.808610	0.283066	0.487805
C	−0.802102	0.291285	−0.656249
C	−2.623216	−1.027530	−0.123251
C	−1.456524	−1.048994	−0.774946
H	−3.323558	−1.843484	−0.050294
H	1.726275	−0.360839	−2.026788
H	−1.586900	2.073017	0.514614
H	1.765734	−2.232823	1.873932
H	−1.025791	−1.874297	−1.315746
H	2.712642	−2.369135	−0.640796
H	−3.667669	0.571207	1.071528
H	−0.758960	0.764529	−1.642660
H	0.183711	−0.133984	2.068347
Cl	1.288108	1.890130	−0.003175

IM14
0 1

C	−2.621453	1.909615	0.316122
C	−3.734629	0.984616	0.363830
C	−3.585542	−0.328450	0.141729
C	−2.266861	−0.902829	−0.110544
C	−1.143104	0.079851	−0.430152
C	−1.402729	1.489780	−0.020913
H	0.088441	0.474610	1.852806
C	0.720443	−0.126179	1.207007
C	2.001819	−0.557241	1.750533
C	0.191841	−0.430736	0.023713
C	3.210067	−0.292894	1.242892
C	2.746400	1.254013	−0.626100
C	3.560732	0.498821	0.081168
O	2.068291	1.935474	−1.267938
H	4.066927	−0.690304	1.765818
H	1.962863	−1.095850	2.687296

H	−4.711110	1.375070	0.611658
O	−2.069520	−2.093827	−0.094948
H	−0.567348	2.172896	−0.073591
H	−2.797378	2.946644	0.555508
H	−4.403224	−1.025747	0.233731
H	−1.131001	0.093663	−1.527818
H	4.584993	0.530973	−0.256581
Cl	0.991457	−1.469726	−1.093026

IM15
0 1

C	2.317260	−1.675136	−0.345827
C	3.252439	−0.605094	−0.067827
C	2.835423	0.639034	0.202615
C	1.401666	0.924202	0.294461
C	0.507963	−0.012436	−0.498330
C	1.019037	−1.417257	−0.502673
C	−1.287990	−0.131010	1.273159
C	−2.121373	−1.165719	1.379626
C	−0.984512	0.105762	−0.176369
C	−2.467812	−1.653686	0.041082
C	−1.825068	−0.925920	−0.870559
H	−3.152579	−2.461181	−0.159053
O	0.978440	1.849200	0.943798
H	0.313393	−2.207977	−0.706155
H	−0.856720	0.468153	2.054446
H	4.306801	−0.838293	−0.033064
H	2.687105	−2.686097	−0.415197
H	−2.502057	−1.579902	2.298442
H	3.510063	1.424743	0.503635
H	0.638184	0.344638	−1.531492
Cl	−1.574943	1.720672	−0.705599
H	−1.879871	−1.005562	−1.942918

IM16
0 1

C	1.940214	−1.975645	0.147507
C	3.096663	−1.431217	−0.249063
C	3.474598	−0.040287	−0.414429
C	2.880787	1.011641	0.111821
C	0.090966	−0.386596	−0.321411
C	0.665142	−1.327499	0.427370
C	−1.979249	−0.319377	1.140435
C	−3.098030	−0.959790	0.804633

C	−1.285585	0.144999	−0.102200
C	−3.242415	−0.957042	−0.654236
C	−2.217070	−0.300790	−1.194205
H	−4.058455	−1.412201	−1.190221
O	2.402008	1.964032	0.551082
H	0.109148	−1.723884	1.267623
H	−1.597587	−0.125013	2.127641
H	3.908880	−2.113177	−0.454333
H	1.944848	−3.047075	0.295471
H	−3.803471	−1.402838	1.488025
H	4.396237	0.200638	−0.921917
H	0.593148	−0.008300	−1.202610
H	−2.020202	−0.110375	−2.234802
Cl	−1.214397	1.957622	−0.103580

IM17

0 1

C	−2.232010	1.492383	0.282619
C	−3.318073	0.546067	0.368432
C	−3.160529	−0.767795	0.153449
C	−1.843550	−1.311747	−0.134193
C	−0.728285	−0.331018	−0.468940
C	−1.017661	1.078113	−0.080993
H	0.111298	−0.783937	2.123747
C	0.922530	−0.647813	1.422160
C	2.151032	−0.354859	1.847887
C	0.627686	−0.869622	−0.016439
C	3.232897	−0.183678	0.903341
C	1.729927	−0.490562	−0.984000
C	3.053625	−0.260051	−0.423707
O	1.504786	−0.427374	−2.173975
H	3.855652	−0.091171	−1.124484
H	4.217197	0.033159	1.292919
H	2.356944	−0.237793	2.900156
H	−4.291419	0.929924	0.636687
O	−1.622121	−2.503011	−0.137091
H	−2.412871	2.531027	0.504712
H	0.533508	−1.957404	−0.144961
H	−3.968581	−1.472894	0.261253
Cl	0.277068	2.202155	−0.212410
H	−0.678726	−0.340973	−1.568224

IM18

0 1

C	−2.568780	0.943227	0.767027
C	−3.322493	−0.292334	0.805227
C	−2.977873	−1.379525	0.102530
C	−1.808145	−1.354820	−0.765770
C	−0.856815	−0.168724	−0.635113
C	−1.432079	1.001956	0.076257
H	0.290168	0.303799	1.839097
C	0.853935	−0.383130	1.218216
C	2.087848	−0.918017	1.780130
C	0.392954	−0.707192	0.014118
C	3.281135	−0.932972	1.175941
C	2.945971	0.281313	−0.977477
C	3.675054	−0.395204	−0.112701
O	2.374822	0.861278	−1.797551
H	4.706211	−0.482275	−0.421029
H	4.105143	−1.372442	1.717843
H	2.038928	−1.297466	2.791207
H	−4.214369	−0.310375	1.414272
O	−1.547902	−2.243731	−1.542756
H	−2.945897	1.810990	1.283029
H	0.904452	−1.451348	−0.582472
H	−3.567644	−2.281904	0.105850
Cl	−0.527357	2.461485	−0.027286
H	−0.591548	0.127930	−1.651788

IM19

0 1

C	−1.361392	1.534725	0.759760
C	−2.468049	0.658207	1.058574
C	−2.648154	−0.524266	0.452147
C	−1.703350	−0.986503	−0.549304
C	−0.434107	−0.174648	−0.782950
C	−0.427658	1.154133	−0.112376
C	0.912284	−1.245619	1.119203
C	2.192832	−1.038896	1.443736
C	0.748541	−1.075341	−0.356125
C	2.944128	−0.693875	0.242645
C	2.113131	−0.673975	−0.804433
H	2.363455	−0.451584	−1.828135
H	4.002113	−0.491204	0.218697
O	−1.862074	−2.013915	−1.171892
H	0.111214	−1.523944	1.784338
H	−3.186885	0.996913	1.790325
H	−1.310158	2.502594	1.230349

H	2.618847	−1.121854	2.430191
H	0.518895	−2.049541	−0.803158
H	−3.495574	−1.158377	0.656687
H	−0.356466	−0.029116	−1.861231
Cl	0.848348	2.225516	−0.533423

IM20

0 1

C	1.930159	1.244264	0.838034
C	3.018231	0.473960	0.736334
C	3.201000	−0.805555	0.081947
C	2.315165	−1.553298	−0.547072
C	0.000726	−0.248746	0.588345
C	0.611991	0.901203	0.318744
C	−2.083633	−0.208506	−0.967968
C	−3.356268	−0.083103	−0.577407
C	−1.292476	−0.837015	0.130845
C	−3.487968	−0.546513	0.797163
C	−2.300332	−0.976995	1.233735
H	−4.407212	−0.545304	1.359797
O	1.616403	−2.293651	−1.094118
H	−1.676640	0.031025	−1.934212
H	3.927345	0.852583	1.179055
H	−2.067244	−1.376521	2.206203
H	2.030060	2.212939	1.304909
H	−4.168079	0.292808	−1.177962
H	−1.051043	−1.854516	−0.210751
H	4.196427	−1.221413	0.027222
H	0.544692	−0.875415	1.286214
Cl	−0.094065	2.157344	−0.651984

IM21

0 1

C	2.273829	1.599210	−0.989211
C	3.377371	0.731586	−0.641902
C	3.285192	−0.203724	0.314220
C	2.047293	−0.405075	1.054542
C	0.920009	0.597217	0.821183
C	1.114645	1.520345	−0.336860
C	−0.843675	−0.935035	−0.097301
C	−2.142496	−1.588746	−0.103815
C	−0.420004	−0.074650	0.823254
C	−3.323615	−0.980906	0.048713
C	−2.844475	1.465912	0.136908

C	−3.649553	0.424198	0.183256
O	−2.208776	2.431015	0.127232
H	−4.197659	−1.614510	0.052113
H	−2.136362	−2.656388	−0.264789
H	4.299636	0.834952	−1.195497
O	1.912146	−1.297462	1.860425
H	0.283193	2.157735	−0.600611
H	2.399526	2.299770	−1.799828
H	4.096017	−0.877320	0.542585
H	0.964804	1.229926	1.714657
H	−4.687115	0.710662	0.271705
Cl	0.216114	−1.421560	−1.385259
H	−1.090067	0.111260	1.649890

IM22

0 1

C	−2.027417	0.202886	−1.831537
C	−3.186422	−0.016180	−0.992893
C	−3.087204	−0.290833	0.316770
C	−1.783774	−0.353299	0.964384
C	−0.570524	−0.407367	0.059052
C	−0.799119	0.053274	−1.335728
C	0.625870	1.736451	0.681643
C	1.728236	2.185642	0.070745
C	0.645842	0.243622	0.719282
C	2.552489	1.058943	−0.347257
C	1.928165	−0.061941	0.023917
H	3.499842	1.123080	−0.854198
O	−1.659210	−0.398661	2.169514
H	0.077079	0.226073	−1.945430
H	−0.169173	2.325251	1.106485
H	−4.164857	0.077032	−1.442191
H	−2.177266	0.501281	−2.857176
H	1.990145	3.217881	−0.092643
H	−3.952606	−0.399296	0.951077
H	−0.353591	−1.483983	−0.007608
Cl	2.452538	−1.670475	−0.231674
H	0.674116	−0.107143	1.753764

IM23

0 1

C	2.242549	−1.037934	−1.474509
C	3.296052	−0.286796	−1.134924
C	3.528378	0.502183	0.061034

C	2.752953	0.657356	1.114548
C	0.300450	−0.170614	−0.233824
C	1.009872	−1.189572	−0.712867
C	−1.444365	−1.657662	0.889705
C	−2.718904	−1.772858	0.503195
C	−0.947770	−0.284452	0.580878
C	−3.167013	−0.528393	−0.106901
C	−2.149564	0.333997	−0.070469
H	−4.147546	−0.339361	−0.508286
O	2.132787	0.871562	2.067103
H	0.644486	−2.198766	−0.566436
H	−0.848421	−2.405968	1.383891
H	4.134036	−0.272367	−1.815569
H	2.325183	−1.618559	−2.382898
H	−3.343160	−2.642522	0.626087
H	4.479108	1.001519	0.176964
H	0.620669	0.842121	−0.447819
Cl	−2.138952	1.942648	−0.648602
H	−0.780295	0.244877	1.528286

IM24

0 1

C	1.671787	−1.352234	−0.128399
C	2.607093	−0.258361	0.028268
C	2.264014	1.032592	−0.085615
C	0.878084	1.392331	−0.339167
C	−0.077920	0.285011	−0.722204
C	0.409009	−1.092717	−0.460851
H	−0.746005	0.917255	1.878995
C	−1.560400	0.483396	1.316270
C	−2.629214	−0.017671	1.935693
C	−1.476213	0.551013	−0.166374
C	−3.730028	−0.563864	1.171210
C	−2.518850	−0.252537	−0.911430
C	−3.702347	−0.658147	−0.166843
O	−2.364390	−0.526846	−2.083651
H	−4.504017	−1.107942	−0.730772
H	−4.593930	−0.925023	1.710551
H	−2.694288	−0.016884	3.012300
H	−0.182485	0.359632	−1.812606
O	0.492243	2.540269	−0.265447
H	−0.294306	−1.900456	−0.603897
H	2.028280	−2.357590	0.023315
H	−1.693721	1.596756	−0.424523

Cl	4.228223	−0.694015	0.402431
H	2.967777	1.832244	0.074734

IM25

0 1

C	−2.052261	−0.630991	1.209233
C	−2.866594	−0.118133	0.125017
C	−2.422511	0.762840	−0.779683
C	−1.057314	1.264849	−0.701268
C	−0.134320	0.675629	0.355976
C	−0.778058	−0.261863	1.309781
H	0.787210	−1.904921	0.192980
C	1.423801	−1.187400	−0.312466
C	2.611454	−1.712337	−0.973336
C	1.067724	0.094575	−0.333998
C	3.853043	−1.225923	−0.868082
C	3.706724	0.620252	0.805653
C	4.357673	−0.134720	−0.055191
O	3.210063	1.338792	1.563716
H	5.414217	0.087737	−0.080019
H	4.628197	−1.720580	−1.433833
H	2.478568	−2.599125	−1.577238
O	−0.647282	2.117725	−1.455607
H	−0.164737	−0.642905	2.113665
H	−2.509080	−1.304967	1.915317
H	0.224588	1.538506	0.927832
H	1.641143	0.808317	−0.910793
Cl	−4.483135	−0.701975	0.051279
H	−3.045220	1.138711	−1.574346

IM26

0 1

C	1.223517	−1.281479	−0.382082
C	2.158227	−0.228763	−0.044859
C	1.807811	1.058291	0.087322
C	0.419765	1.453683	−0.099316
C	−0.524529	0.433747	−0.703659
C	−0.040223	−0.969872	−0.663301
C	−2.116699	−0.010276	1.210584
C	−3.112976	−0.903144	1.183983
C	−1.945780	0.582715	−0.149337
C	−3.655161	−0.973146	−0.168116
C	−2.981553	−0.124511	−0.954181
H	−3.149245	0.055170	−2.003663

H	−4.476019	−1.603859	−0.468263
O	0.032462	2.569972	0.170019
H	−0.760797	−1.741687	−0.895540
H	−1.521287	0.272195	2.062848
H	1.574537	−2.300215	−0.377831
H	−3.472358	−1.481154	2.019497
H	−0.558056	0.721592	−1.763178
H	−2.151555	1.656351	−0.112899
Cl	3.785405	−0.716443	0.228589
H	2.508812	1.817888	0.390647

IM27
0 1

C	−1.343417	−1.186210	−0.334790
C	−2.341122	−0.348009	−0.048187
C	−2.333770	1.097924	0.078980
C	−1.317567	1.921842	−0.077211
C	0.735645	−0.062607	0.363897
C	0.045183	−0.783933	−0.516505
C	2.846785	0.019184	−1.062362
C	3.994237	−0.605301	−0.776286
C	2.152185	0.384034	0.206291
C	4.127946	−0.729862	0.670014
C	3.066637	−0.169498	1.258158
H	4.958215	−1.199660	1.171365
O	−0.500193	2.733637	−0.163769
H	0.543225	−1.138444	−1.410641
H	2.475619	0.271213	−2.041708
H	2.866761	−0.104726	2.314524
H	0.255137	0.225680	1.292227
H	−1.591300	−2.227105	−0.480361
H	4.720176	−0.957557	−1.490888
H	2.165223	1.479573	0.295950
Cl	−3.936526	−1.003013	0.166260
H	−3.267028	1.607785	0.261270

IM28
0 1

C	−1.745440	1.289834	0.513577
C	−1.718692	−0.013242	−0.137529
C	−0.572417	−0.192097	−0.795751
C	0.253769	1.039410	−0.628967
C	−0.592388	1.906873	0.244972
C	2.530573	−0.035067	−0.982150

C	2.997844	−1.096269	−0.315345
C	1.661523	0.775616	−0.078135
C	2.465562	−1.080108	1.042791
C	1.680647	−0.007847	1.192738
H	2.674956	−1.825837	1.792021
H	−0.295676	2.887900	0.577766
H	2.743619	0.231812	−2.004400
H	1.128012	0.274789	2.073061
H	0.362765	1.531864	−1.601790
H	−2.570586	1.657404	1.099457
H	3.663246	−1.849639	−0.704633
H	2.138699	1.747628	0.090438
Cl	−3.020336	−1.124458	−0.018332
H	−0.265203	−1.062747	−1.347479

IM29
0 1

C	3.400273	−0.455356	−1.557717
C	4.176213	−0.897576	−0.415900
C	3.808586	−0.641109	0.846529
C	2.591671	0.106694	1.135878
C	1.721077	0.532473	−0.041054
C	2.254982	0.202687	−1.387806
H	0.104272	−1.130820	−1.513069
C	−0.367111	−0.752998	−0.613282
C	−1.727547	−1.206649	−0.355283
C	0.325669	0.037156	0.203090
C	−2.749799	−0.406933	−0.044745
C	−1.850780	1.898191	−0.257341
C	−2.805590	1.040988	0.040139
O	−1.075034	2.725727	−0.473964
H	−1.933125	−2.261376	−0.460413
H	5.085560	−1.450271	−0.603227
O	2.257389	0.388955	2.264826
H	1.678314	0.535158	−2.239462
H	3.772296	−0.667564	−2.547941
H	4.387441	−0.968769	1.695516
H	−0.096556	0.349198	1.149481
H	1.676312	1.625687	0.037178
H	−3.734785	1.513213	0.317757
Cl	−4.297907	−1.129843	0.266014

IM30
0 1

C	−2.048454	1.980765	0.085347
C	−3.315629	1.302745	0.255574
C	−3.449124	−0.023824	0.105404
C	−2.296191	−0.853380	−0.218290
C	−1.034114	−0.138761	−0.661950
C	−0.971080	1.306057	−0.315236
C	0.537016	−0.766092	1.211826
C	1.781464	−0.304671	1.360602
C	0.221725	−0.910806	−0.241449
C	2.348648	−0.111763	0.033054
C	1.467899	−0.446145	−0.913157
O	−2.337045	−2.064545	−0.178062
H	−0.013682	1.795044	−0.431143
H	−0.157470	−1.017650	1.995763
H	−4.174100	1.892522	0.543837
H	−1.990623	3.035675	0.302843
H	2.306798	−0.103891	2.278451
H	−4.381999	−0.534285	0.285083
H	0.030196	−1.963813	−0.465715
H	−1.092420	−0.191508	−1.758031
Cl	3.946970	0.459293	−0.209342
H	1.618219	−0.408461	−1.978130

IM31

0 1

C	2.476623	−1.839331	0.135316
C	3.626410	−1.314849	−0.303493
C	4.016254	0.074773	−0.460714
C	3.329987	1.164397	−0.184033
C	0.713079	−0.158645	−0.236689
C	1.277356	−1.103009	0.512634
C	−1.096737	0.384144	1.469587
C	−2.392448	0.088303	1.345966
C	−0.504331	0.632000	0.122288
C	−2.723538	0.090348	−0.072479
C	−1.654837	0.395464	−0.808850
O	2.794591	2.172238	0.004965
H	0.805166	−1.380597	1.447404
H	−0.543214	0.474609	2.389102
H	4.420312	−2.002493	−0.553549
H	2.442683	−2.913213	0.257103
H	−3.100508	−0.112124	2.131863
H	5.021517	0.297689	−0.787064
H	−0.237918	1.696600	0.065397

H	1.140011	0.062861	−1.207894
Cl	−4.300686	−0.261542	−0.648149
H	−1.600024	0.463410	−1.880743

IM32

0 2

C	−1.935619	−1.884850	0.195049
C	−2.306214	−0.522940	0.551587
C	−1.435721	0.307484	−0.029283
C	−0.413739	−0.453522	−0.819041
C	−0.856399	−1.868927	−0.595151
C	1.413688	−0.450409	1.035086
C	2.727407	−0.144397	1.110137
C	0.976317	−0.218958	−0.343208
C	3.149531	0.281489	−0.201307
C	2.057037	0.225265	−1.074361
H	4.147211	0.595921	−0.460633
H	−0.337378	−2.715290	−1.010435
H	0.771607	−0.801825	1.825149
H	−3.141251	−0.237117	1.167655
H	2.056517	0.488072	−2.117297
H	−2.467940	−2.761784	0.524753
H	3.358145	−0.200739	1.979468
Cl	−1.420865	2.011648	0.058178
H	−0.489942	−0.186670	−1.874742

IM33

0 2

C	1.617716	−0.610667	−0.951527
C	0.839833	0.319712	−0.123925
C	0.571440	−0.394450	1.143609
C	1.211786	−1.701968	0.998932
C	1.801389	−1.786565	−0.252239
H	1.934817	−0.388384	−1.955194
H	1.181914	−2.477428	1.744692
H	2.299453	−2.659388	−0.641194
C	−2.632521	−0.762805	−0.599396
C	−2.802604	0.490050	0.121241
C	−1.607552	0.901835	0.575514
C	−0.603194	−0.082578	0.170358
C	−1.337127	−1.110146	−0.582019
H	−3.429247	−1.315190	−1.068933
H	−3.741969	0.997978	0.262079
H	−0.884200	−1.981089	−1.020913

H	0.512580	0.117368	2.089794
H	−1.390579	1.795834	1.133423
Cl	1.244838	2.019752	−0.208650

IM34

0 2

C	−1.961386	0.799521	0.000393
C	−0.605035	0.770253	−0.000087
C	−0.686516	−1.701555	−0.000819
C	−2.037012	−1.631116	−0.000363
C	−2.705476	−0.389822	0.000343
H	−2.464672	1.753697	0.000776
H	−2.613695	−2.543808	−0.000576
H	−3.782156	−0.347701	0.000715
C	2.403636	−0.734694	−0.728744
C	2.403121	−0.735147	0.729656
C	1.153802	−0.575465	1.171103
C	0.202681	−0.489952	−0.000341
C	1.154604	−0.574782	−1.170990
H	3.283317	−0.844946	−1.341422
H	3.282511	−0.845780	1.342712
H	0.814395	−0.530562	−2.191389
H	−0.173835	−2.651505	−0.001400
H	0.812891	−0.531921	2.191271
Cl	0.288632	2.242299	−0.000094

IM35

0 2

C	1.442599	2.108560	0.002985
C	1.907392	0.763700	0.220230
C	1.084502	−0.278239	0.048772
C	−0.827711	1.331818	−0.451268
C	0.187156	2.364550	−0.399684
H	2.163534	2.908875	0.066187
H	2.949487	0.591255	0.434841
H	−0.085365	3.355085	−0.732606
C	−2.470031	−1.091121	−0.221411
C	−2.519035	−0.099319	0.742938
C	−1.226863	0.586212	0.808296
C	−0.350533	−0.133275	−0.175399
C	−1.205000	−1.168957	−0.773367
H	−3.306631	−1.701831	−0.518616
H	−3.377342	0.175072	1.332432
H	−0.818365	0.965296	1.732501

H	−1.616887	1.435812	−1.182550
Cl	1.696324	−1.893622	0.094330
H	−0.880796	−1.841558	−1.548361

IM36
0 2

C	2.866094	−0.682033	−0.189231
C	2.427300	0.573684	−0.010720
C	0.085279	−0.295707	0.120817
C	0.617976	−1.585109	0.026535
C	1.969006	−1.788048	−0.087604
H	3.900079	−0.865851	−0.438945
H	3.095651	1.417653	−0.099653
H	2.351790	−2.791464	−0.186537
C	−0.811535	2.375943	−0.191575
C	0.504041	2.177866	−0.018706
C	1.021599	0.846842	0.406790
C	−1.280744	0.011597	0.039493
C	−1.737991	1.296988	−0.076962
H	−1.189839	3.354916	−0.443117
H	−2.796005	1.479040	−0.166529
H	1.060846	0.899513	1.519468
H	−0.056185	−2.425713	−0.013856
H	1.210837	2.988490	−0.115989
Cl	−2.443725	−1.273454	−0.009875

IM37
0 2

C	2.545482	0.475549	0.280483
C	2.511745	−0.889675	−0.170173
C	1.351762	−1.478490	−0.489964
C	0.107663	0.670337	0.152328
C	1.435399	1.228120	0.345692
H	3.444965	−1.403990	−0.338797
C	−2.239657	−1.184862	−0.024055
C	−1.821528	−0.646872	1.178027
C	−0.384104	−0.366266	1.129854
C	0.064256	−0.845082	−0.235105
C	−1.167295	−1.323642	−0.881637
H	0.240304	−0.494840	2.001649
H	1.336937	−2.447147	−0.966838
Cl	−1.031582	1.811812	−0.545211
H	3.502840	0.934696	0.473823
H	−2.446134	−0.407920	2.021514

H	1.495644	2.289332	0.531613
H	−3.261590	−1.419460	−0.271850
H	−1.198393	−1.686186	−1.895223

IM38

0 2

C	3.064419	−0.735201	−0.196453
C	2.204155	−0.114433	−1.022884
C	0.996882	0.210058	−0.266237
C	1.234909	−0.287314	1.090751
C	2.458150	−0.841399	1.120005
H	4.044804	−1.098018	−0.456231
H	2.350405	0.118802	−2.064407
H	2.925574	−1.296773	1.976987
C	−1.650246	1.138057	0.740265
C	−1.534059	0.047597	−0.106730
C	−0.370147	0.188590	−0.978280
C	0.215450	1.498825	−0.603090
C	−0.641387	2.035585	0.458586
H	−2.390508	1.235750	1.516064
H	−0.463824	2.967333	0.966784
H	0.693590	2.148011	−1.318401
H	0.532967	−0.208667	1.901676
Cl	−2.540214	−1.323764	−0.115095
H	−0.378121	−0.204643	−1.981451

IM39

0 2

C	1.545510	−0.082300	0.000009
C	0.327552	−0.668105	−0.000199
C	−0.673195	1.614700	−0.000212
C	0.577258	2.135866	−0.000015
C	1.719736	1.314436	0.000127
H	0.707229	3.207616	−0.000002
H	2.711604	1.733038	0.000267
C	−2.972663	−0.727273	−0.729127
C	−2.972453	−0.727158	0.729548
C	−1.810223	−0.237445	1.169603
C	−0.932231	0.140550	−0.000178
C	−1.810563	−0.237649	−1.169613
H	−3.789461	−1.071293	−1.342413
H	−3.789094	−1.071072	1.343095
H	−1.495088	−0.104199	−2.190280
H	−1.543521	2.253140	−0.000345

H	−1.494447	−0.103803	2.190154
Cl	2.969004	−1.073842	0.000012
H	0.227358	−1.741838	−0.000334

IM40

0 2

C	1.433610	1.353425	−0.138764
C	1.560537	−0.072898	0.012220
C	0.523965	−0.908506	−0.117835
C	−0.987508	1.142961	−0.435206
C	0.245848	1.900487	−0.445630
H	2.331684	1.949397	−0.124222
H	0.199528	2.936693	−0.747165
C	−3.123992	−0.847915	−0.101079
C	−2.870614	0.103247	0.871593
C	−1.453310	0.469964	0.842458
C	−0.839306	−0.399980	−0.213702
C	−1.953645	−1.193835	−0.751940
H	−4.098549	−1.242821	−0.336778
H	−3.596943	0.552094	1.527831
H	−0.903892	0.721961	1.736689
H	−1.853710	−1.894904	−1.563517
H	0.676817	−1.975502	−0.153911
H	−1.779693	1.461618	−1.097648
Cl	3.165367	−0.693543	0.213296

IM41

0 2

C	3.273440	0.201891	−0.240605
C	2.348335	1.154855	−0.044759
C	0.607776	−0.625596	0.160530
C	1.635605	−1.569920	0.041410
C	2.939561	−1.183640	−0.116683
H	4.279177	0.473805	−0.523445
H	2.592918	2.201594	−0.152483
H	3.712021	−1.926810	−0.235845
C	−1.363226	1.373858	−0.136458
C	−0.087279	1.764324	−0.001086
C	0.974518	0.805209	0.420172
C	−0.742970	−0.975509	0.105916
C	−1.705414	−0.005258	0.010905
H	−2.141254	2.077280	−0.387150
H	1.015384	0.890854	1.529839
H	1.374767	−2.618226	0.011502

H	0.186516	2.800785	−0.132461
Cl	−3.369246	−0.442549	−0.081083
H	−1.024421	−2.017222	0.072403

IM42
0 2

C	−1.675224	1.228183	0.267209
C	−0.650875	2.240655	0.227482
C	0.624095	1.944485	−0.058859
C	−0.025484	−0.530142	−0.212689
C	−1.378040	−0.045660	−0.027268
H	−2.700885	1.515934	0.432135
C	2.879304	−0.922341	−0.287260
C	2.135020	−1.299170	0.815817
C	0.958496	−0.434473	0.934710
C	1.085326	0.566198	−0.182380
C	2.314641	0.183633	−0.894403
H	3.759431	−1.434943	−0.639204
H	2.342384	−2.127356	1.472041
H	0.547011	−0.147072	1.890039
H	2.670475	0.679301	−1.781996
H	1.351020	2.728106	−0.210028
H	0.114210	−1.359363	−0.890705
Cl	−2.631622	−1.218341	−0.242631
H	−0.949617	3.268973	0.358282

IM43
0 2

C	−1.988006	−1.743481	−0.220522
C	−0.665164	−1.580056	−0.066724
C	−1.034055	0.882844	0.133934
C	−2.411370	0.624334	0.066374
C	−2.893920	−0.648140	−0.062184
H	−2.379672	−2.710133	−0.499029
H	0.017786	−2.403379	−0.198147
H	−3.955462	−0.820205	−0.143219
C	1.739690	1.285477	−0.178569
C	1.300908	0.031619	0.020779
C	−0.111587	−0.274240	0.407705
C	−0.511816	2.169984	0.004891
C	0.836017	2.384438	−0.129076
H	2.783742	1.459417	−0.385003
H	1.220140	3.382792	−0.262679
H	−1.199379	3.001499	−0.052733

H	−0.080664	−0.369752	1.516672
Cl	2.418789	−1.282471	0.007060
H	−3.090087	1.465109	0.044468

IM44

0 2

C	−2.881846	−0.920831	0.000090
C	−2.605050	0.428362	0.000232
C	−1.213527	0.533593	0.000042
C	−0.603247	−0.790960	−0.000305
C	−1.637133	−1.663851	−0.000170
C	1.641762	−0.603543	1.169137
C	2.741888	0.016355	0.728551
C	0.852646	−1.107904	0.000030
C	2.742217	0.016100	−0.728199
C	1.642185	−0.603807	−1.169000
H	3.514264	0.451241	−1.341377
H	−1.555977	−2.737442	−0.000070
H	1.347471	−0.763655	2.192530
H	−3.299385	1.247531	0.000288
H	1.348208	−0.764080	−2.192458
H	−3.864111	−1.362403	0.000147
H	3.513828	0.451400	1.341935
Cl	−0.353976	1.991599	−0.000210
H	0.933929	−2.200873	0.000124

IM45

0 2

C	−2.705184	−0.754353	0.000089
C	−1.621275	−1.546177	−0.000497
C	−0.413615	−0.715379	−0.000084
C	−0.931750	0.660441	0.000024
C	−2.275464	0.627956	0.000527
H	−3.732405	−1.076734	0.000430
H	−1.596003	−2.623311	−0.000910
H	−2.918725	1.490526	0.000856
C	2.504658	0.115315	0.000561
C	1.892911	−0.387098	−1.132558
C	0.813949	−1.296449	−0.741737
C	0.813566	−1.297032	0.741287
C	1.892404	−0.387539	1.132977
H	3.290483	0.852311	0.000905
H	2.124581	−0.128154	2.151444
H	2.125782	−0.127901	−2.150891

H	0.589905	−2.171386	1.331220
Cl	−0.017351	2.108039	−0.000238
H	0.590151	−2.170144	−1.332539

IM46
0 2

C	−2.115054	−0.069134	−1.216556
C	−0.796667	−0.375756	−1.246727
C	−0.796358	−0.375885	1.246587
C	−2.114795	−0.069235	1.216813
C	−2.805293	0.093978	0.000243
H	−2.648122	0.054872	−2.147430
H	−0.271147	−0.494379	−2.182215
H	−2.647490	0.054685	2.147910
H	−3.853716	0.343821	0.000304
C	2.039364	−1.726127	0.000005
C	2.356846	−0.304948	0.000209
C	1.208449	0.374603	−0.000077
C	0.014785	−0.555600	−0.000187
C	0.714105	−1.894508	−0.000249
H	2.778870	−2.510000	0.000066
H	3.345860	0.119990	0.000506
H	0.166638	−2.821138	−0.000446
Cl	1.009844	2.068378	−0.000058
H	−0.270544	−0.494592	2.181924

IM47
0 2

C	−2.856497	−0.488138	0.214597
C	−2.089468	−1.314865	−0.682905
C	−0.765497	−1.154501	−0.819664
C	−0.885596	0.840467	0.795139
C	−2.268077	0.448187	0.978917
H	−3.898993	−0.723163	0.365452
H	−2.580831	−2.145215	−1.166091
H	−2.815814	0.910345	1.787198
C	1.869010	1.310394	−0.167262
C	0.831724	2.152446	−0.508994
C	−0.431665	1.408647	−0.533766
C	−0.072663	−0.017626	−0.230076
C	1.379450	0.023150	−0.023439
H	2.894075	1.597699	−0.006715
H	0.912191	3.210594	−0.690495
H	−0.168367	−1.873006	−1.360756

H	−1.211655	1.629700	−1.246400
Cl	2.291167	−1.356731	0.383931
H	−0.344775	1.208508	1.655698

IM48
0 2

C	1.506173	−0.070334	1.428554
C	0.086247	−0.243768	1.190266
C	0.807086	−1.211888	−1.072819
C	2.071160	−0.865162	−0.792783
C	2.426154	−0.280823	0.474016
H	1.810989	0.202942	2.427662
H	−0.528609	−0.520833	2.035663
H	2.859265	−1.110723	−1.487610
H	3.469620	−0.110480	0.689943
C	−2.615346	−0.674476	0.087822
C	−2.107214	0.593705	0.289651
C	−0.648067	0.564336	0.149422
C	−0.305096	−0.859337	−0.201405
C	−1.598306	−1.558034	−0.226885
H	−3.654663	−0.940604	0.186625
H	−2.655314	1.483722	0.545729
H	−1.702420	−2.611876	−0.423857
Cl	0.122979	1.941949	−0.586042
H	0.573747	−1.770606	−1.966481

IM49
0 2

C	3.047647	0.269954	0.000538
C	2.769240	−1.140689	−0.000523
C	1.425399	−1.289134	−0.000959
C	0.827294	0.051513	−0.000236
C	1.838730	0.982117	0.000732
C	−1.272772	−0.448475	1.174619
C	−2.057185	−1.432294	0.732452
C	−0.649637	0.250646	−0.000135
C	−2.057713	−1.432476	−0.731251
C	−1.273439	−0.448903	−1.174228
H	−2.616374	−2.120931	−1.343342
H	1.719499	2.049479	0.001345
H	−1.050519	−0.173440	2.190527
H	3.506508	−1.923649	−0.000882
H	4.031566	0.709631	0.001093
H	−2.615366	−2.120685	1.345062

H	0.858017	−2.204298	−0.001715
H	−1.051963	−0.174091	−2.190376
Cl	−1.080397	1.987319	−0.000457

IM50
0 2

C	2.917903	−0.211635	−0.000234
C	2.411933	1.141761	−0.002346
C	1.062621	1.073530	−0.001385
C	0.684614	−0.339030	0.001396
C	1.842909	−1.098453	0.002032
C	−1.502073	−0.299774	−1.167733
C	−2.552719	0.403969	−0.730021
C	−0.723691	−0.819244	0.002559
C	−2.553245	0.408164	0.726748
C	−1.502924	−0.293087	1.169231
H	−3.291346	0.900038	1.339021
H	1.897615	−2.172822	0.003884
H	−1.216121	−0.477342	−2.190402
H	3.016658	2.031492	−0.004271
H	3.960855	−0.483368	−0.000401
H	−3.290406	0.892333	−1.345607
H	0.359355	1.889064	−0.002467
H	−1.217738	−0.464534	2.193162
H	−0.730837	−1.912059	0.005597

IM51
0 2

C	−2.917838	−0.211543	0.000017
C	−2.411938	1.141756	−0.000842
C	−1.062592	1.073528	−0.000644
C	−0.684537	−0.339093	0.000333
C	−1.842725	−1.098442	0.000724
C	1.502543	−0.295291	1.168763
C	2.552866	0.406991	0.727875
C	0.723769	−0.819433	0.000859
C	2.552799	0.405458	−0.728888
C	1.502460	−0.297796	−1.168173
H	3.290500	0.895215	−1.343349
H	−1.897526	−2.172808	0.001441
H	1.217074	−0.468821	2.192259
H	−3.016637	2.031505	−0.001515
H	−3.960780	−0.483322	0.000086
H	3.290689	0.897961	1.341219

H	−0.359324	1.889063	−0.001135
H	1.216597	−0.473346	−2.191215
H	0.730567	−1.912263	0.002059

IM52
0 2

C	2.482201	0.000100	−0.728772
C	1.220352	−0.000089	−1.168283
C	0.263464	−0.000187	−0.000058
C	1.220190	−0.000192	1.168272
C	2.482109	−0.000002	0.728935
H	3.368529	0.000286	−1.342305
H	0.878415	−0.000082	−2.189294
H	3.368343	0.000093	1.342591
C	−2.635698	0.000185	0.000058
C	−1.925938	−1.216528	−0.000043
C	−0.571286	−1.245245	−0.000125
C	−0.570973	1.245127	−0.000037
C	−1.925593	1.216761	0.000037
H	−3.713600	0.000368	0.000147
H	−2.472955	2.147870	0.000063
H	−0.032882	2.181286	−0.000060
H	−0.033502	−2.181579	−0.000218
H	−2.473473	−2.147538	−0.000074
H	0.878154	−0.000290	2.189255

IM53
0 2

C	−2.576028	0.204162	−0.180390
C	−1.546975	1.001140	−0.648712
C	−0.267796	0.526175	−0.102774
C	−0.624551	−0.651831	0.755414
C	−2.082274	−0.760370	0.680545
H	−3.610717	0.304307	−0.464945
H	−1.636134	1.820236	−1.342777
H	−2.659724	−1.514226	1.188678
C	2.442099	−0.412361	0.009137
C	2.100221	0.943201	0.357108
C	0.849657	1.406223	0.212723
C	0.115530	−0.932240	−0.537729
C	1.527187	−1.252255	−0.507657
H	3.479955	−0.707047	0.042764
H	1.839657	−2.185022	−0.954427
H	−0.487974	−1.374169	−1.318303

H	0.636458	2.459060	0.324193
H	−0.077839	−0.853839	1.663863
H	2.893900	1.619646	0.634961

IM54
0 2

C	2.404941	0.722214	−0.090634
C	1.229424	1.417153	0.024871
C	0.000032	0.756505	0.126189
C	1.257298	−1.398833	−0.011444
C	2.392436	−0.704091	−0.190727
H	3.338185	1.252373	−0.197276
H	1.233434	2.496905	−0.027193
H	3.307635	−1.218247	−0.443318
C	−2.392398	−0.704178	−0.190719
C	−2.404878	0.722256	−0.090742
C	−1.229544	1.417182	0.024879
C	0.000000	−0.716969	0.412320
C	−1.257325	−1.398852	−0.011268
H	−3.307647	−1.218256	−0.443272
H	−1.241225	−2.475342	−0.102727
H	0.000142	−0.785715	1.525020
H	−1.233514	2.496942	−0.026989
H	−3.338165	1.252304	−0.197480
H	1.241248	−2.475287	−0.103121

IM55
0 2

C	3.643412	−0.348408	−0.224273
C	2.946741	−1.233076	0.680739
C	1.646306	−0.866932	0.688780
C	1.494627	0.268069	−0.221234
C	2.732144	0.561014	−0.763268
C	−0.391863	1.525753	0.778769
C	−1.600612	0.999350	0.989310
C	0.200106	0.957538	−0.477303
C	−1.880094	0.065990	−0.092131
C	−0.862926	0.017581	−0.955421
H	2.948919	1.343411	−1.468959
H	0.122410	2.240265	1.398288
H	3.398807	−2.033724	1.238936
H	4.697868	−0.389735	−0.443033
H	−2.277439	1.206013	1.800583
H	0.839656	−1.307335	1.250242

H	−0.784289	−0.590248	−1.838834
H	0.355685	1.755153	−1.207880
Cl	−3.345215	−0.817945	−0.185479

IM56
0 2

C	−3.371760	0.438700	−0.000162
C	−2.642747	−0.691772	0.000130
C	−1.229522	−0.323627	0.000052
C	−1.201295	1.141422	0.000006
C	−2.471142	1.579482	−0.000019
H	−4.447174	0.500679	−0.000212
H	−3.008037	−1.705222	0.000164
H	−2.784443	2.610057	−0.000104
C	1.721336	−0.204229	0.000044
C	1.039200	−0.572626	−1.143843
C	−0.209065	−1.218325	−0.741250
C	−0.209096	−1.218281	0.741306
C	1.039160	−0.572452	1.143849
H	1.340992	−0.366192	2.155119
H	1.341142	−0.366749	−2.155153
H	−0.640720	−2.013185	1.327369
H	−0.307081	1.738853	−0.000014
Cl	3.197387	0.674424	−0.000026
H	−0.640683	−2.013202	−1.327391

IM57
0 2

C	−1.100772	−1.224373	−0.000014
C	0.251487	−1.244439	−0.000023
C	0.251468	1.244554	−0.000017
C	−1.100841	1.224446	−0.000008
C	−1.796060	0.000066	−0.000002
H	−1.661059	−2.146336	−0.000016
H	−1.661075	2.146445	−0.000007
C	3.303586	−0.000080	0.728784
C	3.303608	−0.000067	−0.728740
C	2.042040	0.000007	−1.168964
C	1.086241	0.000076	−0.000013
C	2.042005	0.000002	1.168969
H	4.189942	−0.000104	1.342104
H	4.189982	−0.000083	−1.342034
H	1.699792	0.000062	2.189847
H	0.785934	2.182377	−0.000024

H	1.699861	0.000070	−2.189854
H	0.786010	−2.182218	−0.000035
Cl	−3.513292	−0.000080	0.000011

IM58
0 2

C	−1.666555	−0.032898	−0.034171
C	−1.175665	1.270324	0.329979
C	0.126461	1.562844	0.210230
C	0.565633	−0.841189	−0.552003
C	−0.874974	−0.985403	−0.550103
H	−1.892306	2.028379	0.600764
H	−1.308459	−1.861747	−1.006490
C	3.374423	−0.063329	−0.158435
C	2.752740	−0.965679	0.686584
C	1.319628	−0.671937	0.751411
C	1.127376	0.551373	−0.097890
C	2.461896	0.864643	−0.626535
H	4.415913	−0.094678	−0.433536
H	3.221945	−1.793040	1.191496
H	0.741470	−0.811547	1.652078
H	1.113577	−1.346319	−1.334720
H	2.661673	1.670132	−1.313209
Cl	−3.381548	−0.264886	0.032341
H	0.466728	2.579387	0.339420

IM59
0 2

C	−2.923220	−1.159073	−0.245878
C	−1.670948	−1.555019	0.034138
C	−0.974135	0.841153	0.130612
C	−2.314401	1.185450	−0.075759
C	−3.280698	0.224845	−0.222038
H	−3.671092	−1.886818	−0.522352
H	−1.394277	−2.598619	0.001418
H	−4.303433	0.511052	−0.409620
C	1.697303	−0.009221	0.050249
C	0.763078	−0.958664	0.192829
C	−0.645704	−0.574823	0.497348
C	0.067203	1.772780	0.064095
C	1.379589	1.383884	0.059805
H	2.176846	2.103820	−0.020247
H	−0.177389	2.819548	−0.045921
H	−0.716369	−0.598834	1.609400

H	−2.569066	2.229985	−0.185871
H	1.022937	−2.005281	0.163135
Cl	3.355497	−0.440161	−0.206020

IM60

0 2

C	−1.884670	1.423673	−0.059111
C	−1.956841	−0.009981	0.015883
C	−0.691774	−0.576700	−0.174342
C	0.173795	0.476532	−0.365184
C	−0.586595	1.722269	−0.291429
C	2.219368	−0.927844	−0.868614
C	3.193011	−1.199822	0.007081
C	1.646001	0.416835	−0.572396
C	3.315089	−0.094841	0.949777
C	2.422351	0.849950	0.639067
H	4.019458	−0.058244	1.764608
H	−0.163343	2.705677	−0.408487
H	1.888866	−1.547038	−1.685549
H	2.253625	1.782654	1.149639
H	−2.717994	2.093352	0.048838
H	3.800945	−2.089568	0.024008
H	1.903121	1.095269	−1.396653
Cl	−3.389994	−0.872871	0.293024
H	−0.453193	−1.623720	−0.162201

IM61

0 2

C	1.674421	1.434925	0.000397
C	0.390071	1.829716	−0.000023
C	−0.463478	0.645465	−0.000455
C	0.440067	−0.505023	−0.000758
C	1.687798	−0.014252	−0.000212
H	2.553690	2.055045	0.000958
H	0.022950	2.842404	−0.000183
C	−2.679499	−1.334403	0.000065
C	−2.381381	−0.600508	−1.134868
C	−1.821976	0.693459	−0.740529
C	−1.821210	0.693044	0.741328
C	−2.380268	−0.601384	1.135106
H	−3.053353	−2.345248	−0.000100
H	−2.483899	−0.931723	2.154308
H	−2.486022	−0.929866	−2.154271
H	−1.981217	1.580283	1.332138

H	−1.982545	1.581159	−1.330540
H	0.142978	−1.537200	−0.001169
Cl	3.141185	−0.927123	−0.000085

IM62

0 2

C	−1.474114	−0.019082	1.409008
C	−0.148708	−0.020852	1.556043
C	0.532128	−0.002742	0.207117
C	−0.646647	0.009839	−0.735775
C	−1.769189	0.000270	−0.017368
H	−2.223321	−0.029634	2.182167
H	0.408032	−0.033423	2.477491
C	3.357098	0.005932	−0.440412
C	2.666240	1.220497	−0.261588
C	1.347124	1.245677	0.045382
C	1.347239	−1.246427	0.012689
C	2.666449	−1.212961	−0.293377
H	4.406421	0.009192	−0.686577
H	3.199862	−2.142220	−0.428211
H	3.199401	2.153095	−0.372193
H	0.823383	−2.184186	0.121798
H	0.823117	2.180166	0.179107
Cl	−3.373769	0.008344	−0.621034
H	−0.548545	0.024254	−1.806314

IM63

0 2

C	−3.259548	−0.300367	−0.152423
C	−2.874007	1.023089	0.266847
C	−1.592946	1.418962	0.240850
C	−0.927963	−0.937867	−0.523339
C	−2.353031	−1.177920	−0.617269
H	−4.311165	−0.535438	−0.212856
H	−3.649562	1.735199	0.503156
H	−2.677217	−2.080187	−1.114984
C	1.761837	0.038110	0.095234
C	1.164947	−0.927537	0.883044
C	−0.282940	−0.733658	0.834496
C	−0.503613	0.484399	−0.015261
C	0.835003	0.908149	−0.442955
H	1.673199	−1.718950	1.405181
H	−1.331319	2.453081	0.408142
H	−0.912513	−0.921914	1.690554

H	−0.288251	−1.395205	−1.265084
H	1.044029	1.726563	−1.109501
Cl	3.449786	0.115570	−0.218821

IM64
0 2

C	−1.817521	−1.345623	0.811413
C	−0.619921	−0.532149	0.881868
C	−1.884046	1.084429	−0.652325
C	−2.867202	0.178930	−0.760137
C	−2.825179	−1.064651	−0.032905
H	−1.896319	−2.180716	1.491991
H	−0.083012	−0.503290	1.819657
H	−3.758671	0.420106	−1.318430
H	−3.683746	−1.717030	−0.078011
C	1.583440	1.385525	0.454265
C	1.596855	0.115075	−0.096599
C	0.241121	−0.364296	−0.351016
C	−0.637988	0.788719	0.044474
C	0.281453	1.832306	0.526947
H	2.456620	1.914468	0.796205
H	−0.042803	2.781228	0.919616
H	−1.992429	2.070766	−1.078309
H	0.008040	−0.983609	−1.203174
Cl	2.981544	−0.839500	−0.370909

1−MCN
0 1

C	2.044871	−1.712838	0.000128
C	0.691126	−1.545169	−0.000044
C	0.127117	−0.252756	−0.000005
C	2.383872	0.660927	0.000389
C	2.902574	−0.599674	0.000347
C	0.445772	2.174246	0.000257
C	−0.903326	2.362124	0.000089
C	−1.773170	1.259615	−0.000130
C	−1.266277	−0.005427	−0.000174
C	0.989113	0.872994	0.000215
H	−2.840720	1.407409	−0.000264
H	3.036200	1.521619	0.000556
H	3.971343	−0.747413	0.000481
H	0.036423	−2.400828	−0.000213
H	2.460319	−2.708616	0.000095
H	1.119281	3.018151	0.000425

H	−1.316453	3.358601	0.000122
Cl	−2.371554	−1.337245	−0.000449

2−MCN
0 1

C	2.971318	−1.152462	0.000105
C	1.662998	−1.538373	−0.000063
C	0.629860	−0.577711	−0.000022
C	2.331786	1.163221	0.000367
C	3.310641	0.212922	0.000322
C	−0.065259	1.751758	0.000237
C	−1.374618	1.374549	0.000070
C	−1.696228	0.006381	−0.000148
C	−0.729611	−0.952188	−0.000195
C	0.971047	0.794881	0.000196
H	2.586315	2.212942	0.000536
H	4.349109	0.505728	0.000455
H	1.401104	−2.586039	−0.000232
H	3.753787	−1.895413	0.000071
H	0.191406	2.800714	0.000406
H	−2.167077	2.104963	0.000101
H	−0.997009	−1.997272	−0.000365
Cl	−3.364072	−0.449617	−0.000363

Naphthalene
0 1

C	2.412733	0.703727	−0.000019
C	1.234528	1.390861	0.000151
C	0.000025	0.707961	0.000110
C	1.234476	−1.390868	−0.000282
C	2.412710	−0.703725	−0.000238
C	−1.234529	−1.390862	−0.000151
C	−2.412732	−0.703736	0.000019
C	−2.412707	0.703723	0.000238
C	−1.234484	1.390872	0.000282
C	−0.000022	−0.707953	−0.000110
H	−1.230784	2.471145	0.000451
H	−3.350718	1.237255	0.000372
H	1.230772	−2.471142	−0.000450
H	3.350715	−1.237265	−0.000371
H	1.230605	2.471148	0.000320
H	3.350792	1.237175	0.000015
H	−1.230583	−2.471149	−0.000320
H	−3.350796	−1.237172	−0.000015
